# Unraveling the Tether: Exploring Representative Protein Linkers and Their Structural and Thermodynamical Properties

**DOI:** 10.1021/acs.jpcb.4c04194

**Published:** 2025-04-06

**Authors:** Josef Šulc, Jiří Vondrášek

**Affiliations:** † Institute of Organic Chemistry and Biochemistry of the Czech Academy of Sciences, Prague 6 166 10, Czech Republic; ‡ Faculty of Science, Charles University, Albertov 2038, Prague 128 00, Czech Republic

## Abstract

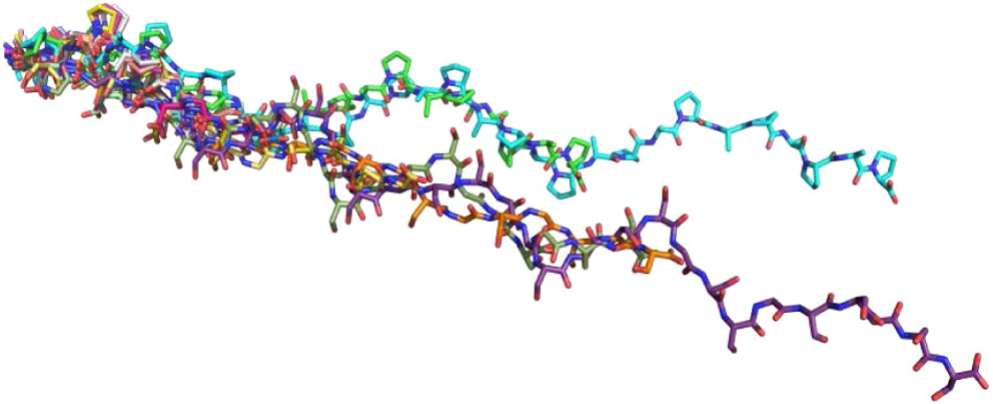

This study explores the thermodynamic and structural behaviors of linker peptides, short polypeptide segments that often bridge protein domains. We are focusing on three prototypical classes—glycine-serine (GS), glycine–glycine (GG), and alanine-proline (AP)—and exploring their conformational dynamics as isolated entities outside a multidomain protein context. Using extensive molecular dynamics (MD) simulations and free energy perturbation (FEP) analyses, we characterize the free energy landscapes, entropic properties, and solvation energetics of 20 representative linkers. Our results reveal a pronounced linear relationship between linker length and key thermodynamic contributions, including zero-point vibrational energy (ZPVE), potential energy, and entropy. Notably, vibrational entropy emerges as a dominant stabilizing term. We also found that AP linkers display more rigid, yet extended conformations compared to the highly flexible GS and moderately flexible GG linkers. These findings underscore the nuanced role of linker composition in contributing to multidomain protein architecture and dynamics, and highlight how thermodynamic forces shape linker conformational behavior. Collectively, our work enhances the mechanistic understanding of protein linkers, offering valuable insights for the rational design of peptide-based systems and informing future efforts to modulate interdomain flexibility and stability in multidomain proteins.

## Introduction

Proteins are biological macromolecules essential to all living organisms, exhibiting a remarkable diversity in their structures and functions. At certain organizational levels, proteins consist of distinct modular units known as protein domains within a single linear chain of amino acids. Higher organizational levels can be characterized by an oligomeric state in which protein components are noncovalently bound to each other. Domains are defined as structurally or functionally independent units within the multidomain protein context, contributing to its wide range of functions.

Protein linkers are polypeptide sequences that connect different protein domains without being part of any domain.^1^ In most cases, domains fold independently of their surroundings and can carry functions that are modulated only by changes in their compositions and contexts.^2,3^ Sequence-based domain definition/identification primarily results from multiple sequence alignments. The discovery of homologous sequences across different proteins has led to the establishment of protein classification databases, such as Pfam.^4^ The structural classification of domains, to some extent, is an extension of Pfam, as represented by the CATH database, which organizes and identifies protein domains based on their structures and provides information on their evolutionary relationships.^5^

To summarize, we consider an amino acid sequence that is not part of a domain defined by MSA or structural classification as a linker.^4^ In this study, we explore the nuanced world of protein linkers, aiming to elucidate their physical characteristics using state-of-the-art computational methods on representative examples.

The spectrum of properties and behaviors of linkers can be simplified into two extremes based on their conformational preferences, categorizing major linker properties into two classes: rigid and flexible linkers. These can be defined based on their propensity to assume defined three-dimensional structures. Among these, Gly-rich linkers are typically considered representatives of flexible linkers, while Pro-rich linkers are viewed as a representative of rigid linkers due to the conformational constraints introduced by proline residues.^6^

Linkers influence the functional outcomes of multidomain proteins.^7−9^ Examples of these effects include guided domain separation, the potential to find preferable interaction interfaces, and modifications of protein folding pathways.^1,10^ The thermodynamic consequences of a linker’s length and its composition may impact the energy landscape of the complex, for example, by enabling or preventing large-scale global interdomain movements and affecting dynamical behavior.^11^

The empirical foundation, rooted in experimental methods, is crucial for understanding the role of protein linkers within the architecture of multidomain proteins. The structural and dynamic behavior of these linkers can be investigated using techniques such as NMR spectroscopy, FRET, SAXS, and others.^12−14^ However, elucidating the behavior of multidomain proteins or the thermodynamic ensembles of their connecting linkers primarily relies on computational methods, especially Molecular Dynamics (MD) simulations. These techniques provide a complementary means to dissect the complexities of protein linkers at the atomic scale, allowing for the exploration of protein/linker dynamics, structural fluctuations, and other thermodynamic parameters, thus bridging experimental observations with theoretical insights.

An important characteristic of peptides serving as linkers is their resemblance to intrinsically disordered proteins (IDPs, i.e., they lack a fixed stable three-dimensional structure) due to their generally limited length and composition, necessitating careful model selection for accurate representation. Despite ongoing advancements and significant improvements that have been made to parameter sets and water models, modeling flexible peptides accurately still remains a challenge for current methodologies.^14−17^ Even with these methods, differences have been observed in the generated ensembles of IDPs across force fields and water models.^18^ To somewhat mitigate this issue, we’ve decided upon using two different, state-of-the-art force fields along with optimized water models.

This study seeks to support the hypothesis that variations in linker properties affect their structure and dynamics, contributing to the diversity and adaptability of multidomain proteins, as indicated by a parameter defining the linkers’ influence on these proteins’ properties.

Our objective is to clarify the dynamics and structural preferences of linkers outside the context of a multidomain protein. To achieve this, we employ several complementary computational methods. Initially, we focus on the significance of entropy in influencing the static and dynamic behavior of linkers, considering their length and composition. We explore the Rigid-Rotor Harmonic-Oscillator (RRHO) model, which calculates translational, vibrational, and rotational entropies for a structure at its energy minimum.^19^ This model is applied under vacuum conditions at an MD level of theory.^20^ To estimate the upper limit of total entropy, we utilize the Schlitter approach, which employs covariance matrices of atomic positions from MD trajectory sets.^21^ This method allows us to approximate absolute entropies alongside standard RRHO entropies, facilitating comparisons across different linker lengths and compositions.

The general way in which entropy can be approximated from atomic fluctuations in time is described by the following two equations, where x represents atomic position of an atom at a point in time, **M** represents the mass matrix and **1** represents the unity matrix.^22^
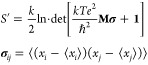


Concurrently, we assess solvation energies to determine their impact on the thermodynamic properties of linkers and their correlation with entropy. Solvation energies are calculated using an accurate explicit method that considers the atomistic resolution of water molecules.^23^ For a precise approximation of solvation free energies across the entire structural ensemble, we employ the Free Energy Perturbation method combined with the expanded ensemble algorithm.^24^ This approach is chosen for its robustness and accuracy in depicting linker solvation energies at the ensemble level.

The aim of this study is to delineate the interplay between entropy, solvation, and dynamic properties, alongside their trends across three distinct classes of linkers and varying lengths. We regard linkers as crucial components of multidomain proteins, significantly influencing their structural and dynamic behavior.

## Materials and Methods

### Structure Generation

Three families of linker structures were generated in PyMol and their N-termini/C-termini were capped using *N*-methyls/C-acetyls so the peptide is uncharged on both termini.^25^ These three kinds of linker peptides were based on GG, GS and AP-mers. The overview of these structures can be found below in Table 1.

**Table 1:**
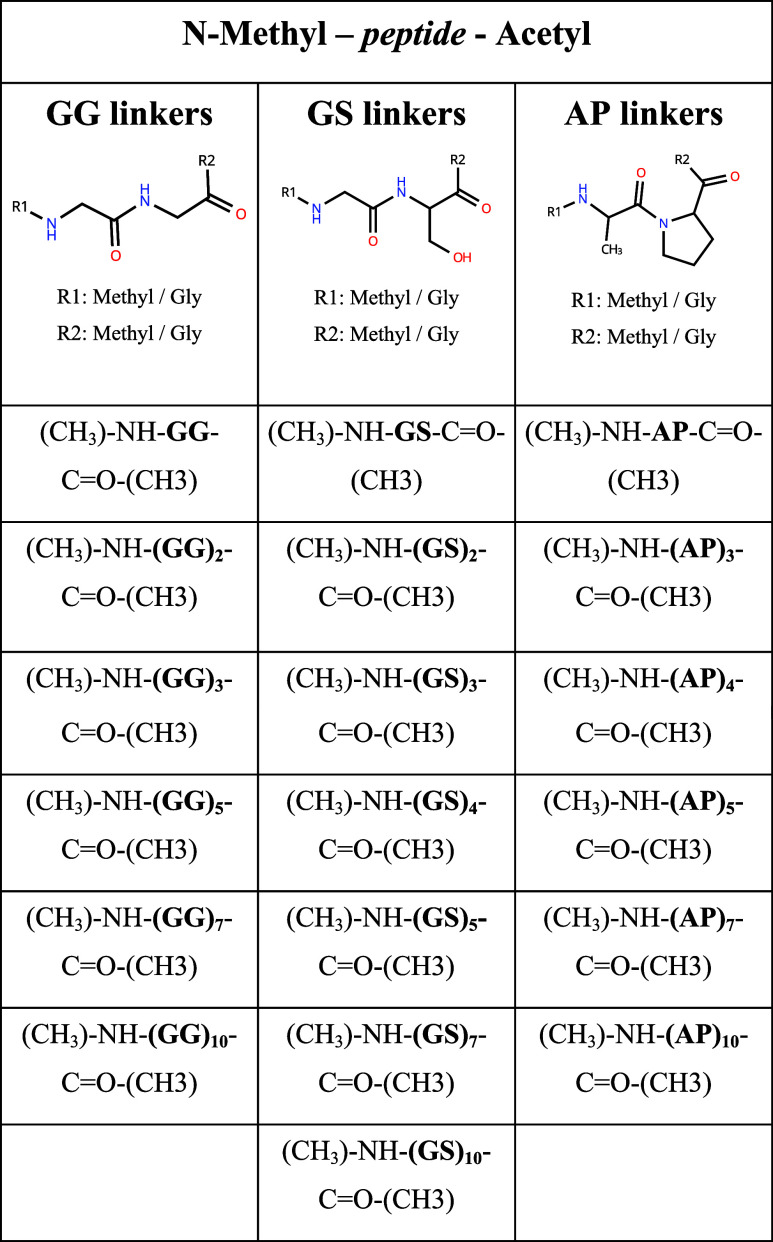
Peptide Structures Used in This Study^a^

a The peptides consisted of dipeptide unit (GG/GS/AP) repetitions, indicated by subscript integers. N-termini were capped with *N*-methyl, while C-termini were capped by acetyl residues.

All of the structures were optimized using the semiempirical quantum chemistry package XTB.^26^ This approach uses the tight-binding density-functional, where the standard DFT formulation is simplified by expanding the electron wave functions in terms of atomic orbitals.^26^ The peptide linker geometry was optimized using the dispersion corrected self-consistent GFN2-xTB2 functional with *extreme* optimization criteria setting. Geometry optimizations were stopped, when energy convergence reached Δ*E* < 5 × 10^–8^ Eh (Hartree units) and geometry convergence Δ*G* < 5 × 10^–5^ Eh**·**α^–1^ (Hartree per Bohr radius).^23,27−29^ ALPB implicit solvation model was used, and the electronic temperature was set to 300 K.^30^

### MD Simulations

MD simulations and subsequent steps were conducted using two distinct combinations of force fields and solvent models: CHARMM36m + TIP3P Charmm and ff14SB + OPC water.^15,16,33^ GROMACS version 2021.3 was utilized for the MD simulations, built in the standard mixed-precision mode.^31,32^

For the CHARMM36m (July 2022) simulations, each linker structure (19 in total) minimized by XTB was immersed in TIP3P-Charmm water within dodecahedral boxes, ensuring a padding of at least 1.7 nm. All cut-offs were set to 1.2 nm.

Sodium and chlorine ions were added to a total concentration of 150 mM NaCl in each system. Bond lengths involving hydrogens were constrained using the LINCS algorithm, and water molecules were constrained using SETTLE, employing a 2 fs time step. With PME-type electrostatics, all structures underwent minimization in two steps (Steepest Descent followed by Conjugate Gradient) and were then equilibrated in the NVT ensemble at 300 K using the V-Rescale thermostat (with separate coupling for the linkers and the rest of the system) and subsequently in the NPT ensemble at 1 bar pressure using the Parrinello–Rahman barostat. Both equilibration phases lasted for 100 ps each. Production NPT MD trajectories of 3000 ns for all peptides in water were conducted in three replicates, each starting with differently assigned velocities from the same equilibrated structure. Convergences for the three CHARMM36m replicates were assessed by comparing the similarity of backbone RMSD (root mean-squared deviation) distributions to verify if the simulation time was sufficient to explore the conformational landscape. ff14SB RMSDs were compared to these.

For the ff14SB + OPC-water based runs with Li/Merz ions, GROMACS was employed, utilizing a set of parameters converted from the AMBER simulation suite, adapted for GROMACS.^33−35^ The linkers were immersed in a box under identical conditions as before but solvated with OPC water instead. For ff14SB, cutoffs were set to 1.0 nm. The minimization and equilibration steps were then simulated analogously to the CHARMM36m runs. Production simulations of 3500 ns were calculated, with one replicate each.

Topologies were modified by removing any nonlinker atoms, and energies were recalculated using the trajectories to obtain linker-only potential energies.

### Free Energy Surfaces

Distance between the centers of mass of the terminal capping groups was taken as the collective variable to plot the free energy surfaces of the linkers.

For the *CHARMM36m* three independent replicas, concatenated trajectories (9000 ns in total) were used to construct the surfaces, while only the single trajectory was used for each of the *ff14SB* trajectories, based on 3500 ns simulation time being considered to likely be sufficient. Distances as a function of time were analyzed using the *pyreweighting* script, with bin sizes of 0.05 nm and no reweighting.^36,37^

To quantify the structural flexibilities of each of the peptides, we used MDAnalysis Python Library^38^ to calculate persistence lengths of the peptides, using concatenated trajectories for CHARMM36m and the single trajectory for ff14SB. To establish sampling efficiency, distance/time functions were analyzed for autocorrelation times.

### Schlitter Entropy

Schlitter’s formula is utilized to calculate absolute entropies from MD trajectories by leveraging atomic position fluctuations to compute a mass-weighted covariance matrix. This atom-positional fluctuation matrix facilitates an approximation of an upper bound to the true absolute entropy. In this approach, the first six vectors of the matrix are disregarded to isolate translational and rotational entropy from the absolute entropy upper bound approximation.^21,39^ Trajectories were corrected for periodic boundary conditions and adjusted for rotation and translation. Covariance matrices of atomic positions over time were computed and diagonalized using the GROMACS *gmx covar* and *gmx anaeig* modules. Schlitter entropy was then calculated at *T* = 300 K. For the CHARMM36m three replica runs, averages and standard deviations were derived for the trio.

### Normal Mode Analyses

To discern zero-point vibrational energy (ZPVE), vibrational, rotational, and translational entropies, the rigid-rotor harmonic-oscillator (RRHO) model was applied, utilizing normal-mode analysis to assess vibrational frequencies, from which ZPVE and vibrational entropy was approximated. Starting structures underwent minimization using a double-precision version of GROMACS 2021.3. Initially, the steepest descent (SD) algorithm reduced the maximum force F below 1000.0 kJ/mol/nm. Subsequently, minimization continued with the conjugate gradient (CG) algorithm, interspersing every thousandth step with an SD step, aiming for a convergence criterion of *F* < 0. This essentially entailed minimizing until the change in energy, represented in double-precision floating-point format, fell below detectable thresholds, utilizing the maximum capabilities of the computer code. Upon reaching stationary points, mass-weighted Hessian matrices were calculated using inbuilt GROMACS tools for harmonic analysis and RRHO entropy estimation.

### Solvation Free Energies

To rigorously calculate solvation free energies, an alchemical cycle was employed, systematically decoupling peptide electrostatic and van der Waals interactions in distinct steps and substituting VdW interactions with soft-core interactions. This necessitated simulating systems both in vacuo and in solvent within NVT ensembles. The linkers were gradually decoupled from the solvent using a discrete auxiliary λ variable, transitioning from λ = 0 (the starting system) to λ = 1 (the system with all peptide-rest interactions turned off, including peptide self-interactions). A second set of simulations was then conducted *in vacuo*. Following standard protocols, electrostatic interactions were disabled first, succeeded by the substitution of dispersions with Beutler soft-core interaction potentials to preclude singularities, setting Sc_alpha = 0.5, Sd_power = 1, Sc_sigma = 0.3.^40^

The Expanded Ensemble approach, complemented by the Wang–Landau algorithm, was utilized to balance the histogram in expanded λ-space, arranging thermodynamic λ-states to ensure completion of calculations.^41^ The arrangement of thermodynamic λ-states was selected for each calculation in a way in which the calculation could proceed until completion.

**Figure 1:**
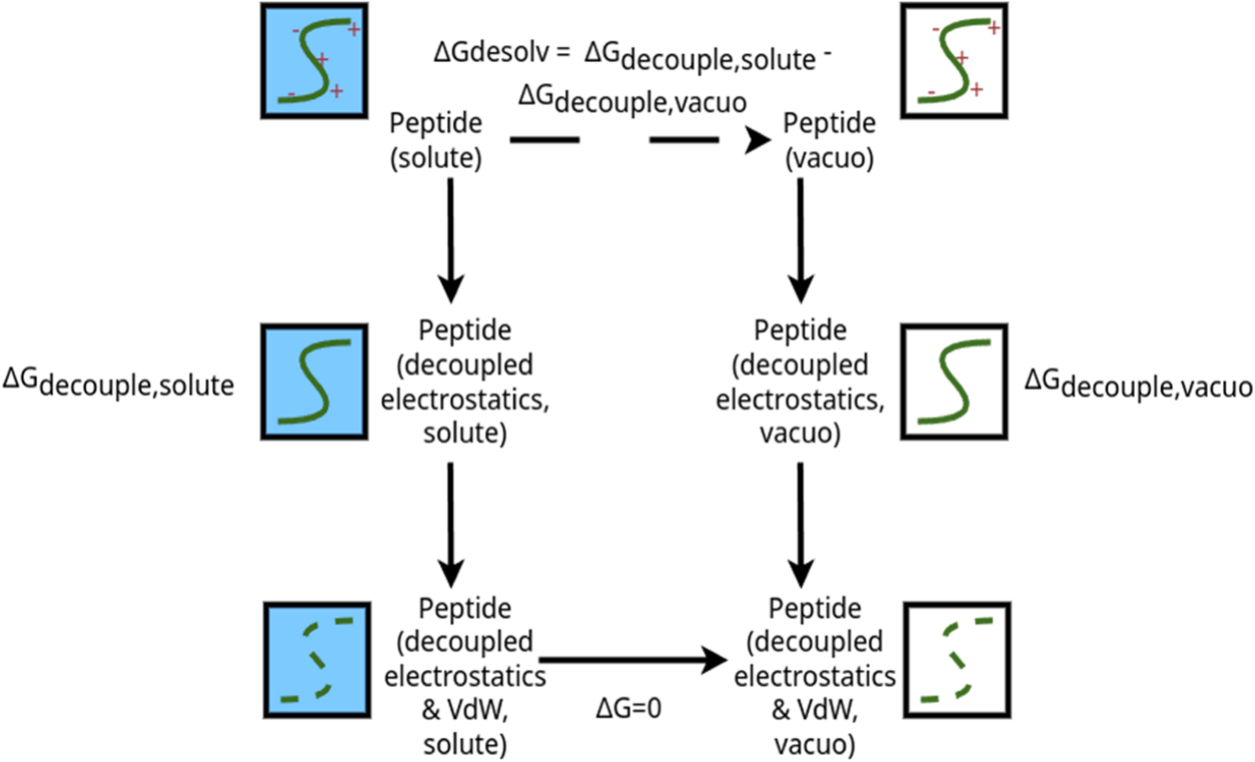
Thermodynamic cycle for calculating molecule solvation free energy in the alchemical perturbation methodology, where molecule is discharged in solvent and in vacuo to calculate its solvation energy. (blue) solvent, (green) peptide representation, (red) charges representation.

Starting with WL_delta = 1.0 and scaling down by 0.7 whenever the discrepancy among states fell below 20%, λ-states were deemed equilibrated once WL_delta reached 0.001. Transitioning between λ-states employed the Metropolized Gibbs algorithm, with production data gathered every 2 ps. The resultant *dH/dλ* values underwent analysis using the Multistate Bennett Acceptance Ratio to verify consistency with Thermodynamic Integration (TI) and other methods, employing the *alchemical_analysis* package for analysis.^42^ From the Expanded Ensemble trajectories, samples post-100 ps simulation time were considered. The congruence between TI and MBAR results was evaluated, with MBAR findings adopted as the conclusive values. As depicted in the scheme (Figure 1), calculating Δ*A*_1_ – Δ*A*_2_ yields the solvation Helmholtz free energy of the peptide, denoted as Δ*G* for comparison purposes, despite discussing solvation within an NVT ensemble.

## Results and Discussion

### Structure Generation

Peptides of three types were created by repeating AP, GS, and GG dipeptide units. The AP (XP) was chosen as an assumed representative of a rigid linker, while GS was chosen as a representative of a flexible linker. The repetition of GG was selected because of its significant relevance to many experimental works and as an often-present artificial linker peptide. Because the linker is usually connected to neighboring proteins on both termini, N-termini/C-termini were capped with *N*-methyls/C-acetyls. These uncharged capping groups were added to simulate these peptides being connected by peptide bonds at both ends.

**Figure 2:**
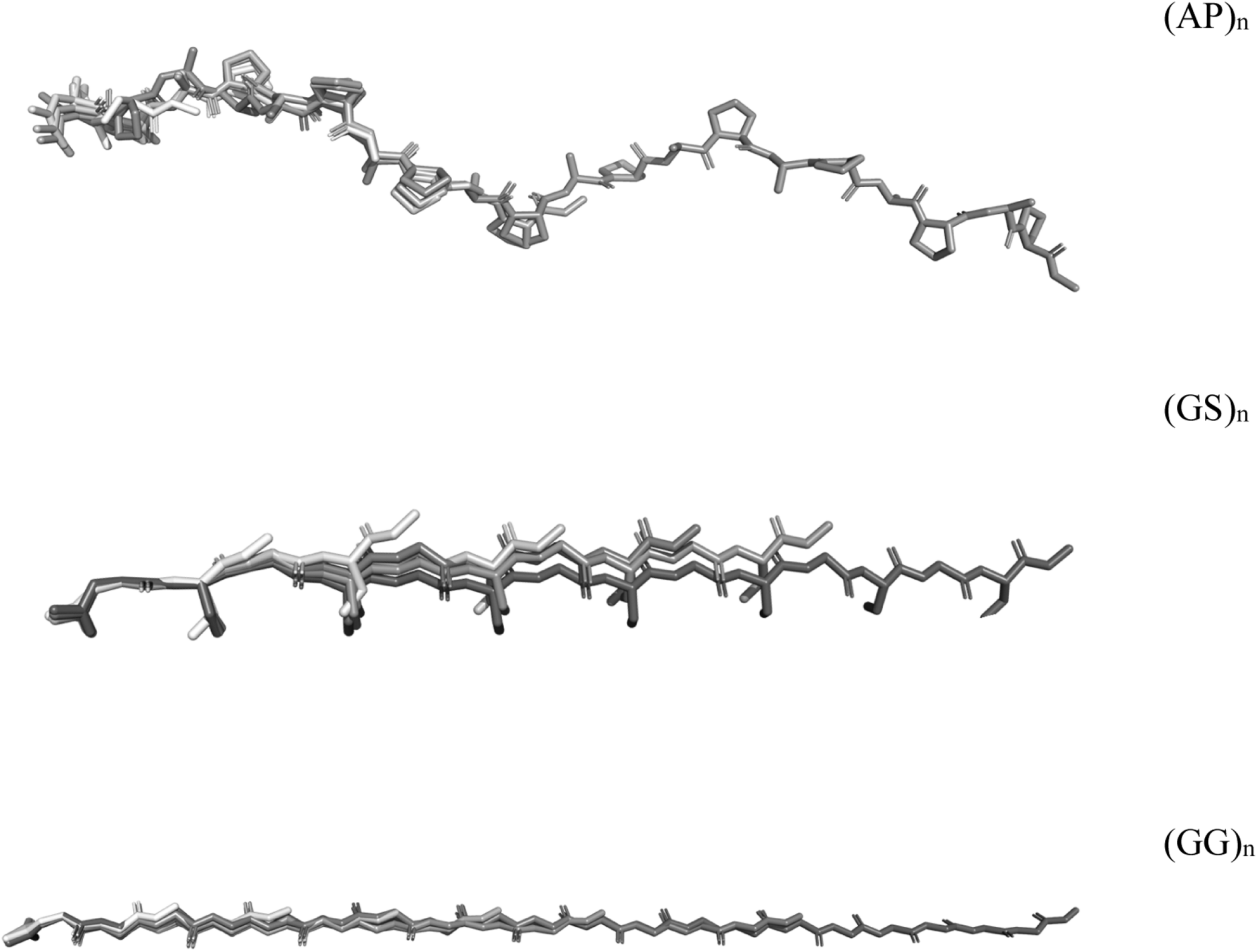
Geometrically optimized structures of AP-, GS-, and GG-based linker peptides in a stick representation excluding hydrogens. Calculated at the GFN-xTB2 level of theory with ALPB-based implicit water solvation. Darker shades indicate increasing linker lengths.

Extended linkers were then optimized using a semiempirical QM, tight-binding DFT approach included in the xTB package. All the optimization steps were carried out using an ALPB implicit water solvent with the electronic temperature set to 300 K. The geometry optimizations were carried out using an *extreme* convergence setting with the more accurate GFN-xTB2 functional. Thus, optimized structures for further steps were obtained. These structures can be seen in Figure 2.

### MD Simulations and Potential Energies

MD trajectories were obtained to capture the thermodynamic behavior of the linkers. Two different force field + water models were selected, guided by the expected IDP-like nature of our peptides. These were CHARMM36m (July 2022 version) with the TIP3P-Charmm water model, and an AMBER-based ff14SB with the OPC four-site water model.^15,16,33^

The CHARMM36m simulations were run in three replicates, targeting at least 3.0 μs per simulation for a combined simulation time of at least 9.0 μs per peptide. To check for convergence between each set of three replicas, RMSD distributions calculated for peptide backbones were compared against each other. Resulting comparisons can be seen in Figure S1. The other set of simulations (ff14SB+OPC) was run in a single replicate with the expectation that this sampling time would be sufficient.

**Figure 3:**
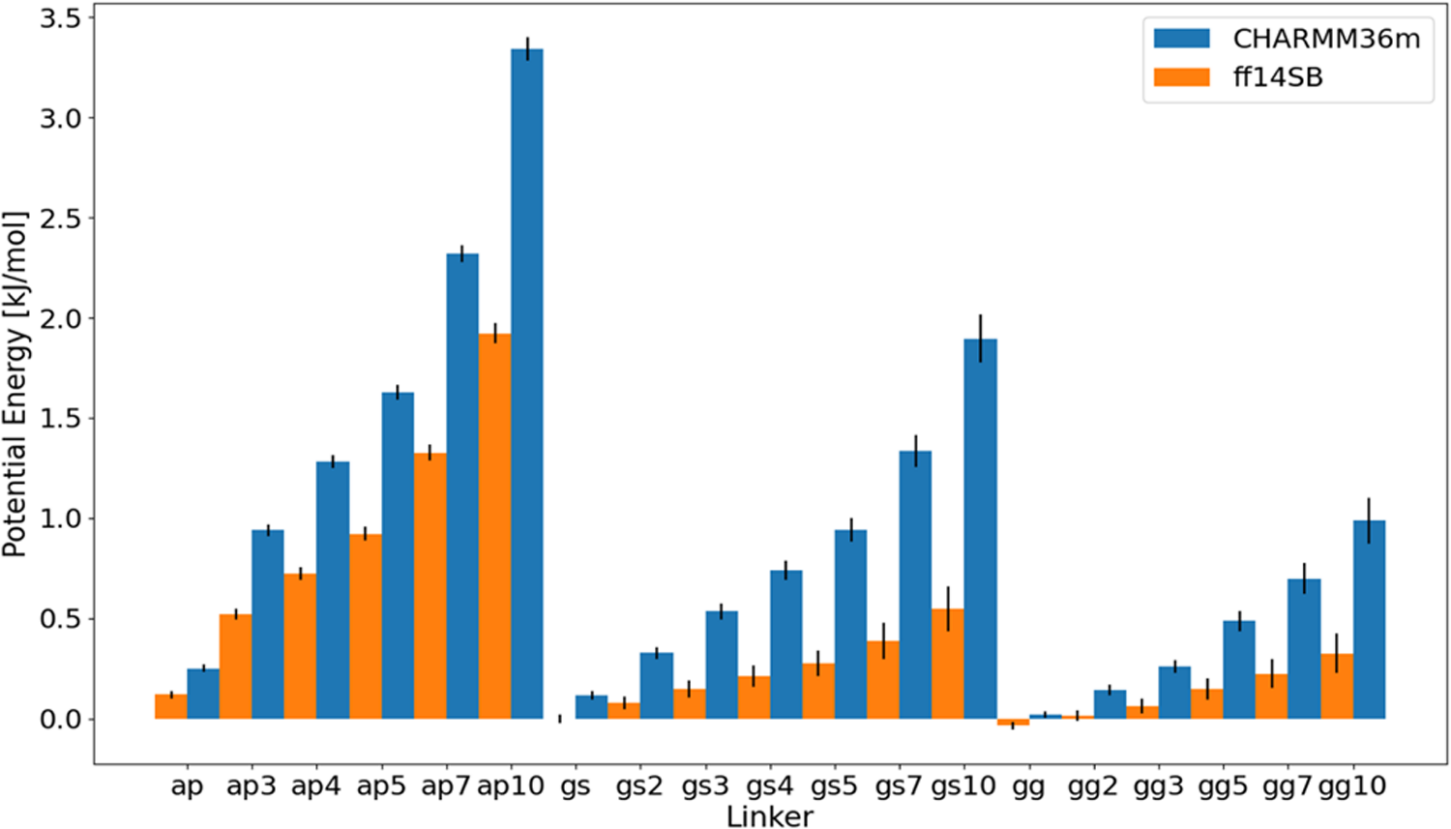
Potential energies of linkers (U) in both utilized force fields (CHARMM36m and ff14SB+OPC), containing only linker–linker interactions (excluding interactions with water). Black lines on bars represent statistical deviations, while the height describes mean potential energy derived from the trajectory.

Using topologies modified to contain only the linker atoms, energies were recalculated using the trajectories to yield potential energies of the linkers only, which are one of the terms of the total linker energies. Omitting solvation energy in this case is ameliorated by the later addition of separately calculated value of Δ*A* of solvation from FEP calculations. These enthalpies, presented as their means and standard deviations, can be found in Table 2 and graphically in Figure 3. The pV term needed to yield enthalpies was considered to be insignificant.

**Table 2:** Potential Energies of Linkers (*U*) in Both Utilized Force Fields (CHARMM36m and ff14SB+OPC), Containing Only Linker–Linker Interactions (Excluding Interactions with Water)^a^

linker	*U* [kJ/mol] CHARMM36m	*U* [kJ/mol] ff14sb+OPC
AP	248.89 ± 17.909	118.76 ± 17.829
(AP)_3_	937.86 ± 28.040	520.00 ± 27.926
(AP)_4_	1282.2 ± 31.939	720.25 ± 31.632
(AP)_5_	1626.9 ± 35.565	920.67 ± 35.041
(AP)_7_	2317.8 ± 43.145	1326.0 ± 41.401
(AP)_10_	3341.8 ± 56.727	1922.6 ± 48.534
GS	115.36 ± 20.669	–2.0180 ± 20.981
(GS)_2_	325.71 ± 30.248	74.984 ± 31.851
(GS)_3_	533.77 ± 38.532	145.28 ± 42.866
(GS)_4_	738.92 ± 47.369	209.73 ± 54.629
(GS)_5_	940.94 ± 57.424	275.43 ± 64.653
(GS)_7_	1336.8 ± 79.927	386.25 ± 88.643
(GS)_10_	1895.0 ± 118.60	547.69 ± 110.87
GG	20.728 ± 16.540	–35.118 ± 18.691
(GG)_2_	141.07 ± 24.276	14.730 ± 26.800
(GG)_3_	259.05 ± 31.523	61.557 ± 35.183
(GG)_5_	486.63 ± 51.053	146.46 ± 53.431
(GG)_7_	697.67 ± 76.513	223.37 ± 70.953
(GG)_10_	985.87 ± 114.56	322.94 ± 98.627

a Shown are means and statistical deviations.

The observed linear growth aligns with our expectations, as the number of interacting atoms increases monotonically with the addition of more dimers.

Given that we are attempting to approximate absolute potential energies, we’re limited to report mostly trends and not quantitative values. In general, the calculated potential energies are larger (sometimes significantly – see (AP)_10_, (GS)_5_, (GS)_7_ and (GS)_10_ cases), while the trends among the two different force fields remain conserved.

### Mapping of the Free Energy Surface and Estimation of Total Entropy

Distances between terminal capping group centers of mass were used as a collective variable to define the structural flexibility of the linkers. To establish if the sampling was sufficient, these distance/time functions for the concatenated trajectories (or single ff14SB trajectories) were analyzed for autocorrelation times. For all the linkers, AC of zero was reached sooner than with a lagtime of 5000 frames (50 ns with sampling every 10 ps) for CHARMM36m and ff14SB. Given that our sampling times exceeded this AC time by orders of magnitude in each of the simulations, we consider our sampling to be sufficient. Autocorrelation functions are present in Supplementary S2.

**Figure 4:**
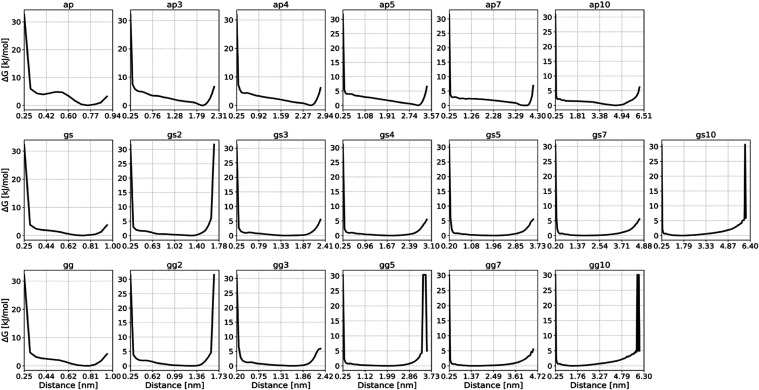
CHARMM36-based PMFs of the distance between (AP)_n_-, (GS)_n_-, and (GG)_n_-based peptide linkers terminal capping groups. Results based on 3 × 3 μs MD simulations for CHARMM36m.

**Figure 5:**
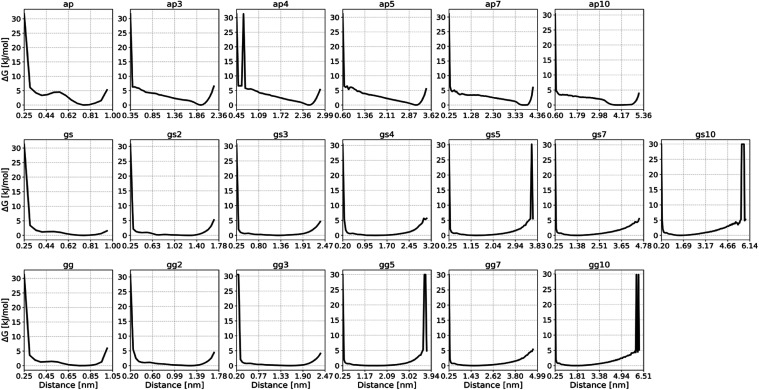
ff14SB-based PMFs of the distance between (AP)_n_-, (GS)_n_-, and (GG)_n_-based peptide linkers terminal capping groups. Results based on 1 × 3.5 μs MD simulation for ff14SB+OPC.

The resulting free energy surfaces are visualized in Figure 4 (CHARMM36m) and Figure 5 (ff14SB).

One readily apparent characteristic of the (AP)_n_-based linkers, compared to the other types, is their propensity for assuming more defined structures, as represented by the rightmost free energy minimum on their free energy surfaces. In contrast, (GS)_n_ and (GG)_n_-based linkers exhibit a high degree of flexibility when considering the distance between their termini as a collective variable to describe structure.

Persistence lengths were also calculated, and are noted in Table 3. For CHARMM36m simulations, *l*_p_ values were calculated for each trajectory. For (AP)_n_ linkers, persistence lengths are higher than for (GG)_n_, which is higher than values of (GS)_n_. This indicates that (AP)_n_ linkers are noticeably more rigid than the other two, while (GS)_n_ seems to be the most flexible of the linkers. It should be noted that the values differ between the two force fields, with ff14SB consistently describing (AP)_n_-based linkers as having longer *l*_p_ and (GG)_n_ and (GS)_n_ linkers as having shorter persistence lengths. Nevertheless, the order between the types remains consistent across the force fields.

**Table 3:** Calculated Persistence Lengths of Linker Peptides^a^

linker	*L*_P_ [nm] (CHARMM36m)	*L*_P_ [nm] (ff14SB+OPC)
AP	(3.93, 3.96, 3.97)	4.25
(AP)_3_	(11.8, 12.1, 12.0)	13.9
(AP)_4_	(13.7, 13.8, 13.9)	16.7
(AP)_5_	(15.6, 15.9, 15.9)	20.2
(AP)_7_	(16.6, 17.0, 16.5)	23.3
(AP)_10_	(15.6, 16.3, 16.8)	26.8
GS	(3.35, 3.36, 3.37)	3.32
(GS)_2_	(4.16, 4.11, 4.14)	3.55
(GS)_3_	(4.30, 4.42, 4.28)	3.42
(GS)_4_	(4.32, 4.33, 4.37)	3.32
(GS)_5_	(4.31, 4.32, 4.29)	3.34
(GS)_7_	(4.23, 4.26, 4.23)	3.34
(GS)_10_	(3.76, 4.07, 3.92)	3.36
GG	(4.14, 4.12, 4.13)	3.82
(GG)_2_	(4.43, 4.40, 4.43)	4.14
(GG)_3_	(4.56, 4.53, 4.49)	4.10
(GG)_5_	(4.46, 4.37, 4.43)	3.98
(GG)_7_	(4.33, 4.28, 4.29)	3.93
(GG)_10_	(4.17, 4.45, 4.10)	3.89

a Two force fields were utilized—CHARMM36m (sampling time 3× 3000 ns) and ff14SB (sampling time 3500 ns). The numbers in brackets indicate values for each of the three replicas simulated with CHARMM36m.

These findings link thermodynamic information to the previously mentioned classification of peptide linkers as rigid or flexible.

Considering the behavior of the linkers in the two different parameter sets (ff14SB+OPC and C36m+TIP3P Charmm), we observed qualitatively identical behavior between both sets. As will be seen later in the work, the major difference between the simulations are absolute values of potential energies, which have no impact upon the final result, however.

Based on the trajectories, eigenvectors of the covariance matrices were calculated for each trajectory, starting from 5 ns until the end. The first six vectors were removed before analysis. At a temperature of 300 K, Schlitter entropies were calculated using the *gmx anaeig* tool included in GROMACS. For CHARMM36m, the results were presented as the average and standard deviation of the three replicas. The results are displayed in Table 4.

**Table 4:** Schlitter Entropies at *T* = 300 K and *p* = 1 bar for a Set of Terminal-Capped Peptides Consisting of AP, GS, and GG Dipeptide Repetitions^a^

linker	-TS_Schlitter_ [kJ/mol] CHARMM36m	-TS_Schlitter_ [kJ/mol] ff14sb+OPC
AP	–73.86 ± 1.851	–75.18
(AP)_3_	–320.4 ± 1.998	–312.7
(AP)_4_	–473.0 ± 2.093	–454.5
(AP)_5_	–624.9 ± 2.879	–591.7
(AP)_7_	–907.5 ± 5.580	–855.9
(AP)_10_	–1374 ± 5.527	–1280
GS	–95.07 ± 0.05388	–101.3
(GS)_2_	–245.4 ± 0.4466	–256.0
(GS)_3_	–391.1 ± 0.7934	–408.9
(GS)_4_	–546.1 ± 0.3896	–567.3
(GS)_5_	–690.9 ± 0.6436	–715.8
(GS)_7_	–993.5 ± 0.8001	–1023
(GS)_10_	–1398 ± 37.05	–1474
GG	–80.04 ± 0.07399	–89.17
(GG)_2_	–171.2 ± 0.1303	–190.9
(GG)_3_	–271.6 ± 0.4293	–296.3
(GG)_5_	–496.0 ± 0.8243	–528.3
(GG)_7_	–716.0 ± 0.7531	–748.8
(GG)_10_	–1004.79 ± 37.29	–1088

a Results based on 3 × 3 μs MD simulations for CHARMM36m and on 1 × 3.5 μs MD simulations for ff14SB.

Given that the Schlitter approach approximates the upper bound of absolute entropy and the first six eigenvectors (which describe translation and rotation) were disregarded, we infer that the resulting Schlitter entropy comprises vibrational and conformational terms. Subsequently, we approximate the upper bounds of conformational entropy by subtracting the calculated vibrational entropies from the values outlined in this section.

### Normal Mode Analysis

Using a double-precision compiled version of GROMACS, structures were minimized to computer precision. Mass-weighted Hessian matrices were then calculated and used to analyze the RRHO entropy. All these runs were conducted in vacuo, due to the inherent limitations in diagonalizing matrices for large systems (i.e., those in solvent). The results of this analysis are summarized in Table 5, while the vibrational contributions to total NMA entropies are detailed in Table 6.

**Table 5:** RRHO Entropies Obtained by Normal Mode Analyses on Geometrically Optimized Structures of Terminally Capped Peptide Linkers Utilizing CHARMM36m and ff14SB+OPC Force Fields

Peptide (*T* = 300 K)	-TS [ kJ/mol]NMA, Charmm36m	ZPVE [ kJ/mol] CHARMM36m	-TS [ kJ/mol] NMA, ff14SB+OPC	ZPVE [ kJ/mol] ff14SB+OPC
AP	–180.3	776.1	–180.4	791.4
(AP)_3_	–343.2	1808	–343.2	1854
(AP)_4_	–424.4	2324	–424.4	2386
(AP)_5_	–505.5	2840	–505.6	2917
(AP)_7_	–667.7	3871	–667.9	3980
(AP)_10_	–910.9	5419	–911.5	5574
GS	–175.0	629.8	–174.7	635.7
(GS)_2_	–254.3	997.8	–257.5	1008
(GS)_3_	–332.4	1366	–334.7	1383
(GS)_4_	–410.5	1734	–411.9	1757
(GS)_5_	–488.7	2102	–489.2	2132
(GS)_7_	–644.9	2838	–643.8	2881
(GS)_10_	–879.1	3943	–875.8	4004
GG	–167.5	543.7	–162.7	548
(GG)_2_	–238.0	827.4	–226.4	836.1
(GG)_3_	–308.5	1111	–290.2	1124
(GG)_5_	–449.7	1678	–417.6	1700
(GG)_7_	–590.8	2246	–545.1	2277
(GG)_10_	–802.6	3097	–736.3	3141

**Table 6:** Vibrational-Entropies Obtained by Harmonic Oscillator Approach with Normal Mode Analyses on Geometrically Optimized Structures of Terminally Capped Peptide Linkers Utilizing CHARMM36m and ff14SB+OPC Force Fields

peptide (*T* = 300 K)	-TS_vibr_ [kJ/mol] Charmm36m	-TS_vibr_ [kJ/mol] ff14SB+OPC
AP	–85.37	–85.34
(AP)_3_	–237.1	–236.9
(AP)_4_	–314.9	–314.7
(AP)_5_	–393.3	–393.3
(AP)_7_	–551.7	–551.8
(AP)_10_	–790.7	–791.1
GS	–80.61	–80.32
(GS)_2_	–153.5	–156.7
(GS)_3_	–227.2	–229.5
(GS)_4_	–301.9	–303.2
(GS)_5_	–377.1	–377.5
(GS)_7_	–528.6	–527.4
(GS)_10_	–757.8	–754.1
GG	–75.07	–70.14
(GG)_2_	–139.6	–127.9
(GG)_3_	–206.2	–187.7
(GG)_5_	–341.9	–309.7
(GG)_7_	–479.2	–433.3
(GG)_10_	–686.8	–620.4

A significant similarity between the calculated vibrational entropies (and RRHO entropies overall) was found between CHARMM36m and ff14SB force fields for both (AP)_n_ and (GS)_n_-based linkers. On the other hand, for (GG)_n_ linkers, ff14SB RRHO entropies are consistently lower, differing by 4.93 kJ/mol for the shortest GG and 66.4 kJ/mol for GG_10_. ZPVE values are consistently somewhat larger for all the studied structures when using ff14SB.

The entropies calculated by the RRHO approach include translational, rotational, and vibrational contributions. Together with the Schlitter entropies, which approximate the upper bound of absolute entropies (incorporating conformational and vibrational terms in our approach), we obtain an approximation of the total linker entropies.

### Solvation Free Energies

The values of solvation free energies, as detailed in Table 7, for both considered force fields, are on the same order of magnitude. Since OPC is a 4-point water model designed to enhance the description of solute–solvent interactions, its comparability with results from a 3-point TIP3P-Charmm model highlights the not-so-significant advantage of using OPC, especially considering the theoretical 25% additional computational cost, caused by the addition of additional particle, compared to TIP3P. Due to technical issues, solvation energy was not obtained for GG_10_ using ff14SB.

**Table 7:** Solvation Free Energies at *T* = 300 K for a Set of Terminally Capped Peptides Consisting of AP, GS, and GG Dipeptide Repetitions^a^

linker	*A*_solv_ [kJ/mol] CHARMM36m	*A*_solv_ [kJ/mol] ff14SB+OPC
AP	–66.72 ± 0.215	–75.14 ± 0.474
(AP)_3_	–168.8 ± 0.893	–176.9 ± 1.607
(AP)_4_	–166.4 ± 2.118	–218.0 ± 1.937
(AP)_5_	–160.9 ± 2.989	–196.4 ± 2.175
(AP)_7_	–254.2 ± 1.538	–221.5 ± 1.404
(AP)_10_	–293.2 ± 3.802	–366.2 ± 9.591
GS	–93.14 ± 0.324	–94.94 ± 0.458
(GS)_2_	–137.9 ± 0.916	–149.5 ± 1.153
(GS)_3_	–169.6 ± 1.893	–169.0 ± 0.87
(GS)_4_	–219.2 ± 0.916	–215.6 ± 1.929
(GS)_5_	–237.4 ± 1.726	–260.2 ± 2.763
(GS)_7_	–312.0 ± 2.822	–304.2 ± 2.522
(GS)_10_	–413.1 ± 3.948	–390.0 ± 20.28
GG	–87.57 ± 0.134	–82.16 ± 0.325
(GG)_2_	–122.7 ± 0.347	–117.4 ± 0.672
(GG)_3_	–154.5 ± 0.763	–150.4 ± 0.973
(GG)_5_	–221.5 ± 1.806	–221.3 ± 2.029
(GG)_7_	–276.0 ± 1.676	–255.7 ± 2.467
(GG)_10_	–400.5 ± 3.247	(N/A)

a Calculated utilizing a standard desolvation thermodynamic cycle, using FEP-EE approach in GROMACS. Shown are means and standard deviations of the analyses.

When considering the total free energy balance, solvation acts as a stabilizing force and is energetically negative, similar to entropies (-TS).

### Linker Thermodynamics

Having obtained all the thermodynamic terms contributing to the Gibbs Free Energy (G), we can now discuss specific terms. For the purposes of this study, we divide the total change in the free energy of a linker into the following terms: the free energy of solvation, potential energy of linker–linker interactions, the pV factor (which is ignored), Zero-Point Vibrational Energy (ZPVE), and entropy. This entropy change comprises RRHO (translational, rotational, vibrational) and Schlitter components, with the vibrational entropy subtracted to yield an upper bound to the conformational entropy.
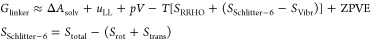
Solvation energies were derived in the form of Helmholtz Energy (*A*) from the NVT ensemble. However, for the purposes of our discussion, we consider this term to be roughly equivalent to the Gibbs Free Energy (*G*), ignoring the pV factor, in a hypothetical NPT calculation.

**Figure 6:**
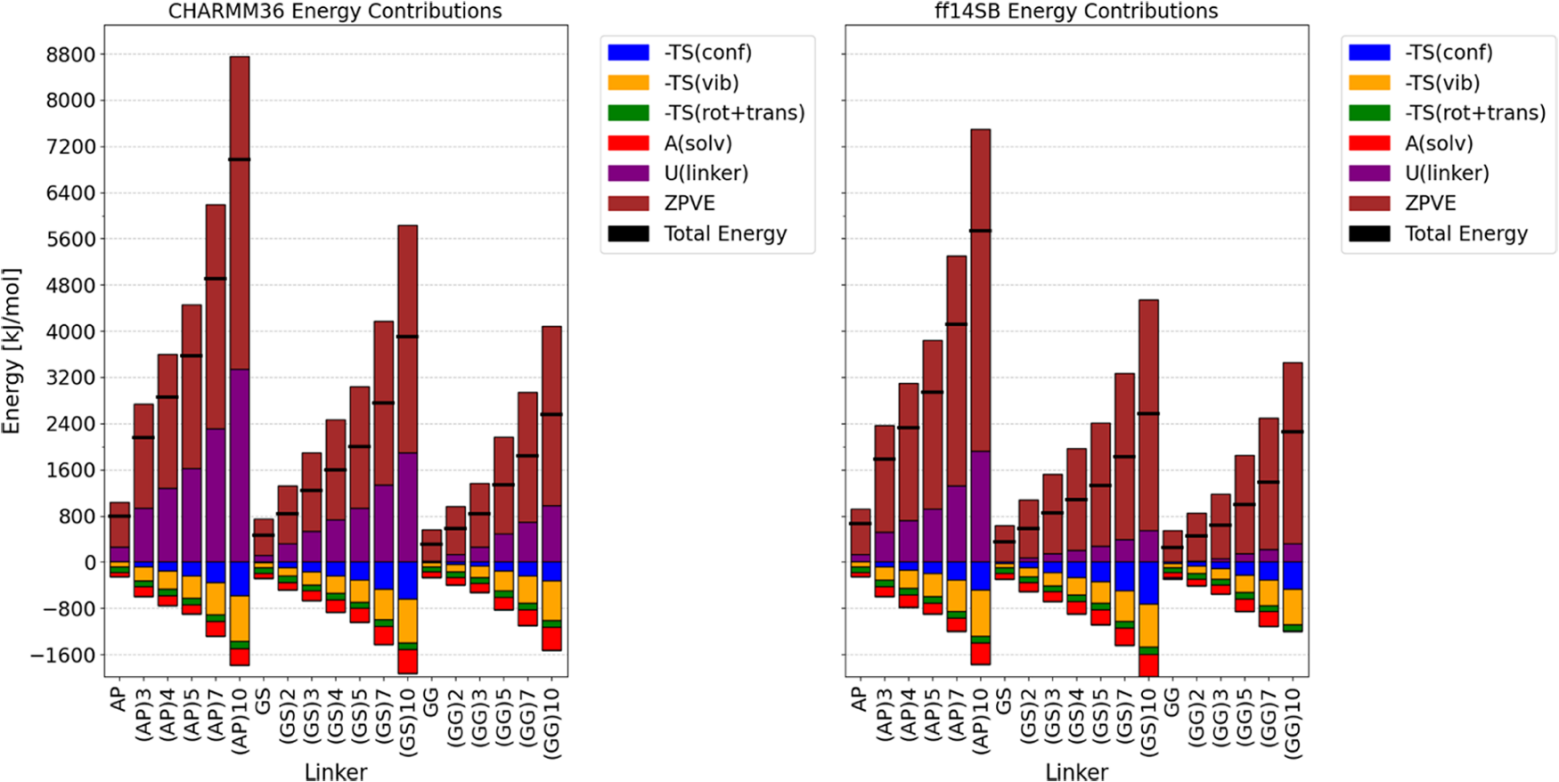
All the absolute thermodynamical contributions to absolute Gibbs Free Energy of a set of peptide linkers. The dashed blue line indicates G = 0, the black horizontal lines indicate the total energy sum. (left) CHARMM36m (right) ff14SB+OPC force field. Results based on 3 × 3 μs MD simulations for CHARMM36m and on 1 × 3.5 μs MD simulations for ff14SB.

Vibrational, translational, and rotational entropies were approximated using the RRHO approach, while conformational entropies were derived from the Schlitter approximation. In this context, the Schlitter approximation includes only vibrational and conformational entropies. By subtracting the vibrational entropies, we obtain an upper bound approximation for the conformational entropies. This is due to the fact that the first eigenvectors of the positional fluctuation matrix, which represent translational and rotational contributions, were not considered in calculating Schlitter Entropies. The total energy balances provided by specific contributions are visualized in Figure 6.

By aggregating all contributions (except for GG_10_ linker using ff14SB, where the solvation term has not been calculated), the “absolute Gibbs energies” turn out to be positive for all linkers, as anticipated. The predominant positive contributor to these energies is the Zero-Point Vibrational Energy (ZPVE) across all types of linkers, followed by the potential energy from linker–linker interactions. Among the negative contributions, conformational entropy (or its upper bound) is the most dominant, exhibiting the quickest increase with the lengthening of the linker. This rapid growth is intuitively expected since each addition of a new subunit augments the conformational possibilities.

**Figure 7:**
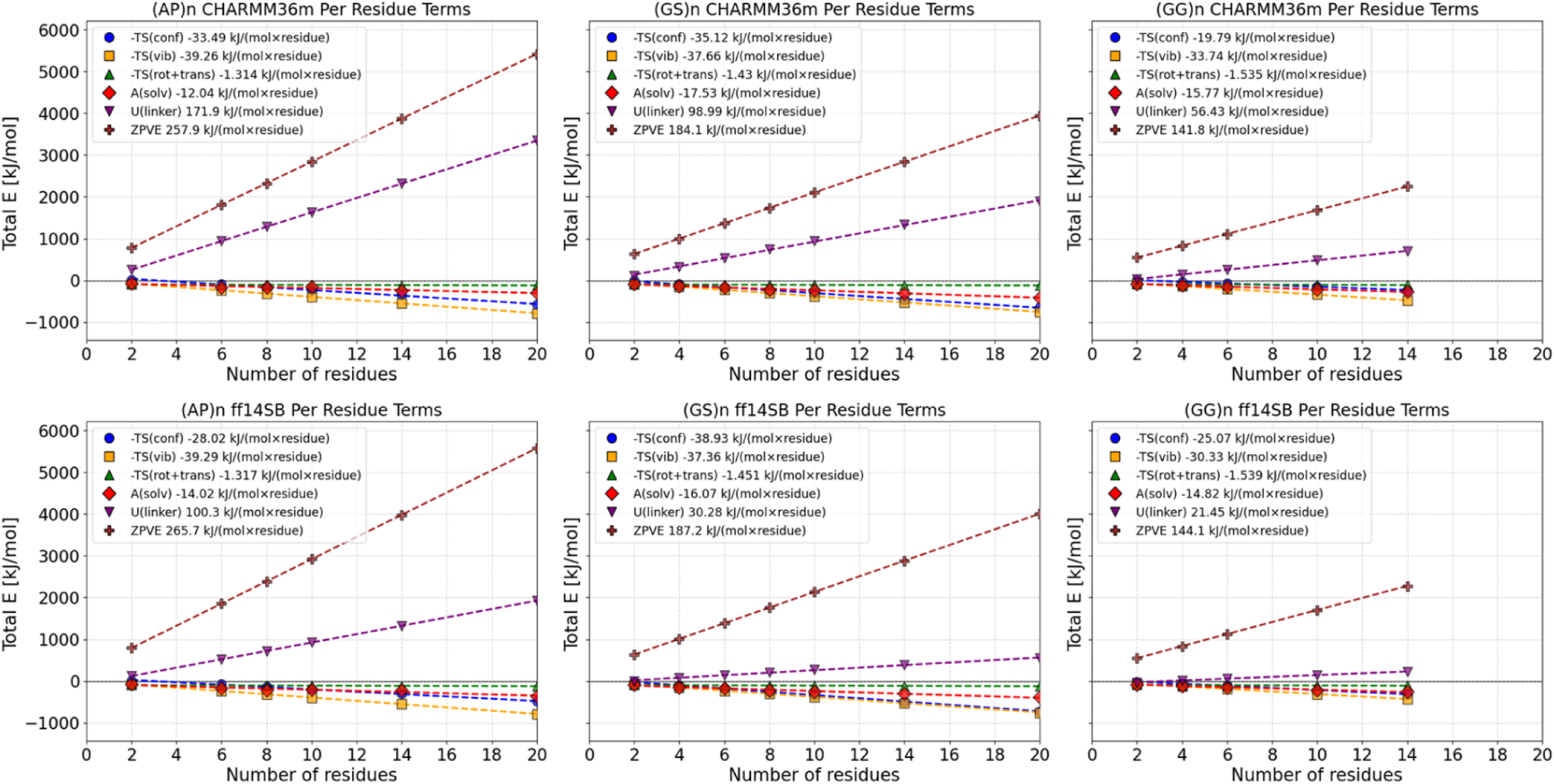
CHARMM36m and ff14SB+OPC per-residue contributions to the total free energy of the linkers, with linear fitting and the calculated slopes (increase in the energy term per “added residue”). Results based on 3 × 3 μs MD simulations for CHARMM36m and 1 × 3.5 μs for ff14SB+OPC. Conformational entropy was calculated using Schlitter approach, RRHO approach was used for the other entropy terms and ZPVE. Solvation energies were calculated using standard thermodynamic cycle, using FEP-EE approach in GROMACS.

To more distinctly analyze the effect of increasing linker length, energies were broken down into per-residue values, showing the energy change brought by the addition of a single residue. For CHARMM36m, these per-residue decomposed energies and the relationship between the number of residues and energy were linearly fitted, resulting in slopes depicted in Figure 7.

As the linker lengthens, all contributions increase linearly, but different terms become dominant in various types of linkers. For (AP)_n_ linkers, the ZPVE and potential energy (U) terms exhibit the fastest growth, both being positive contributions. The most significantly increasing negative term in this context is vibrational entropy, followed by conformational entropy. For all of the linker families, the fastest-growing destabilizing energy term is ZPVE term.

In every scenario, it can be stated that vibrational entropy emerges as the most significant numerically negative term in the total energy equation across all observed cases. This trend is expected to persist for peptides longer than those studied here, with both vibrational and conformational entropy becoming increasingly important as a thermodynamic influence, especially for the more flexible (GG)_n_ and (GS)_n_-type linkers. This linear relationship enables predictions about the contributions of these terms to linkers of varying lengths with these compositions.

For ff14SB + OPC simulations, the observed trends remain consistent, with the only notable differences being the lower slope of potential energy growth (indicating slower growth with linker length compared to CHARMM36m) and a somewhat steeper increase in solvation energy for the (AP)_n_-type linkers.

## Conclusions

This study meticulously analyzed the thermodynamic behavior, solvation energies, and entropy contributions of three distinct types of peptide linkers: (AP)_n_, (GS)_n_, and (GG)_n_. Utilizing sophisticated computational methods, including RRHO and Schlitter entropy calculations, alongside GROMACS simulations under various conditions, we have elucidated the nuanced interplay between the structural flexibility, energetics, and thermodynamics of these linkers.

The investigation revealed that (AP)_n_-based linkers exhibit a propensity for assuming more defined, extended structures compared to the highly flexible (GS)_n_ and (GG)_n_-based linkers. Despite this structural rigidity, characterized also by the increased persistence lengths of (AP)_n_s over the other two types of linkers, all linkers maintained significant flexibility under biologically relevant conditions.

Our analysis further highlighted the pivotal role of Zero-Point Vibrational Energy (ZPVE) as the major positive contributor to the Gibbs energies across all linker types, indicating its significant impact on the overall energetics of the system. Notably, the negative contributions, dominated by vibrational and conformational entropy, underscore the critical role of entropy in defining the thermodynamics of peptide linkers, particularly its rapid increase with linker length due to the enhanced conformational possibilities offered by each additional subunit.

The study also delves into the solvation free energies, revealing comparable values across different force fields and highlighting the nuanced trade-offs between accuracy and computational efficiency in modeling solute–solvent interactions.

In conclusion, this comprehensive analysis sheds light on the complex thermodynamic landscape governing peptide linkers, underscoring the intricate balance between structural characteristics, energetics, and entropy. The findings not only enhance our understanding of peptide linker behavior but also provide valuable insights for the design and optimization of peptide-based systems, with implications for their application in a wide range of biological and technological contexts. The linear relationships identified between linker length and various thermodynamic contributions offer a predictive framework for extrapolating these trends to linkers of different lengths and compositions, paving the way for future explorations and applications in peptide science.

## Data Availability

Simulation parameters and structures referenced in this work are deposited and available on Zenodo (10.5281/zenodo.10931051). Software and parameters sets needed are available as open-source and are referenced in the text.

## Supporting Information Available

RMSD distributions (Figure S1) and autocorrelation functions of end-to-end distances (Figure S2) (PDF)
Set of input .mdp files used for MD simulations with the ff14SB force field (ZIP)
Set of input .mdp files used for NMA analysis in GROMACS (ZIP)
Set of input .mdp files used for MD simulations with the CHARMM36m force field (ZIP)
Extended linker structures in .pdb formats, preoptimization (ZIP)
Set of input .mdp files used for MD/FEP simulations with the CHARMM36m force field for in-solute replicas (ZIP)
Set of input .mdp files used for MD/FEP simulations with the CHARMM36m force field for in-vacuum replicas (ZIP)
All energy terms for all linkers obtained from the three replica trajectories using CHARMM36m (CSV)
All energy terms for all linkers obtained from the single replica trajectories using ff14 (CSV)
