# Supplementary material for: Unraveling the Tether: Exploring Representative Protein Linkers and Their Structural and Thermodynamical Properties
Source: J Phys Chem B. 2025 Apr 6;129(15):3720–30. doi: 10.1021/acs.jpcb.4c04194 (PMC12010332; doi:10.1021/acs.jpcb.4c04194)
Supplement: Supplementary file 1 — jp4c04194_si_001.pdf [file jp4c04194_si_001.pdf]

Unraveling the Tether: Exploring Representative Protein Linkers and Their Structural and Thermodynamical Properties

[1,2]*Josef Šulc*, [1]*Jiří Vondrášek**

[1]Institute of Organic Chemistry and Biochemistry of the Czech Academy of Sciences, Prague 6, Czech Republic

[2]Charles University, Faculty of Science

Supplementary

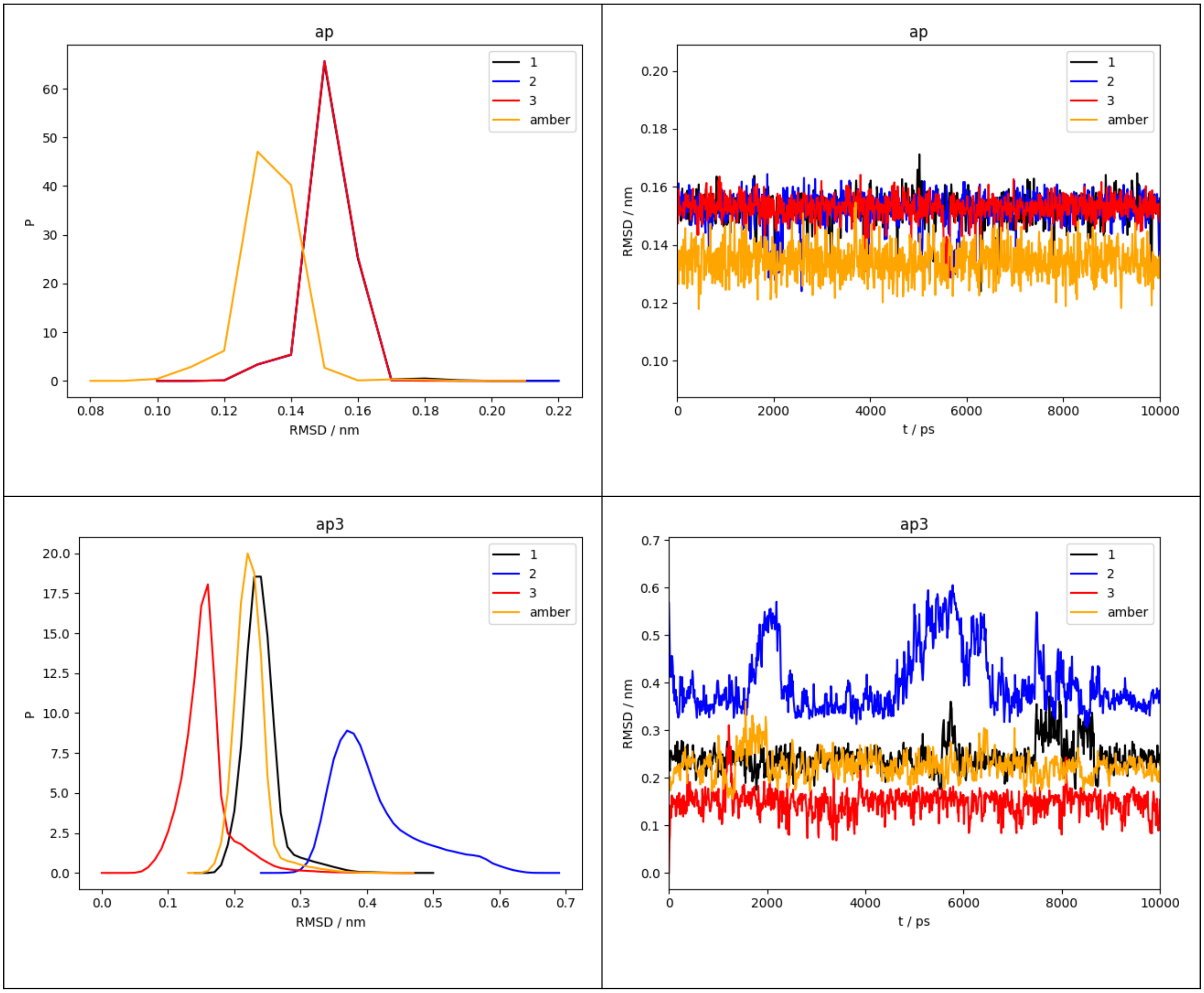

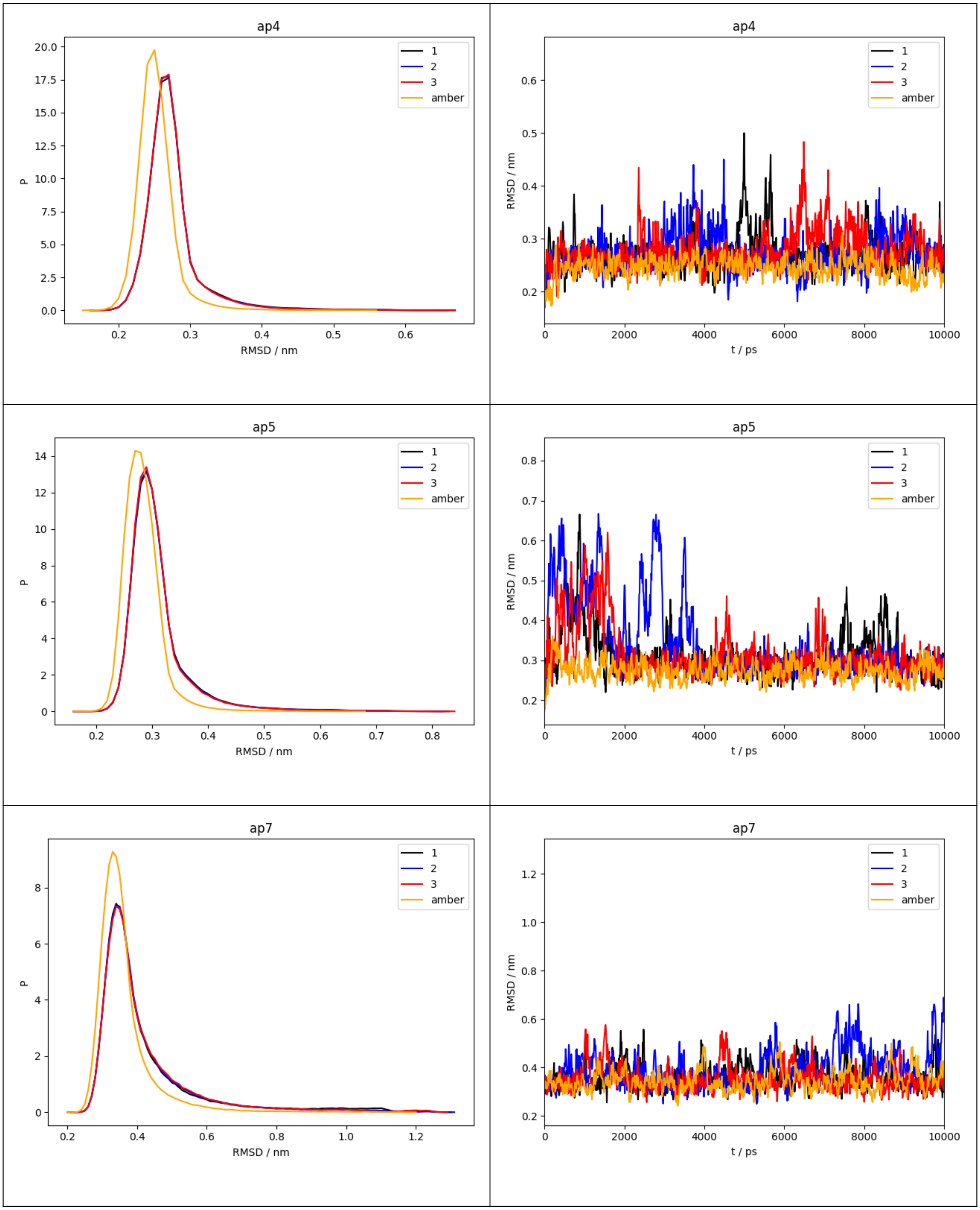
ap4
P
RMSD / nm
1
2
3
amber
ap4
RMSD / nm
t / ps
ap5
P
RMSD / nm
ap5
RMSD / nm
t / ps
ap7
P
RMSD / nm
ap7
RMSD / nm
t / ps

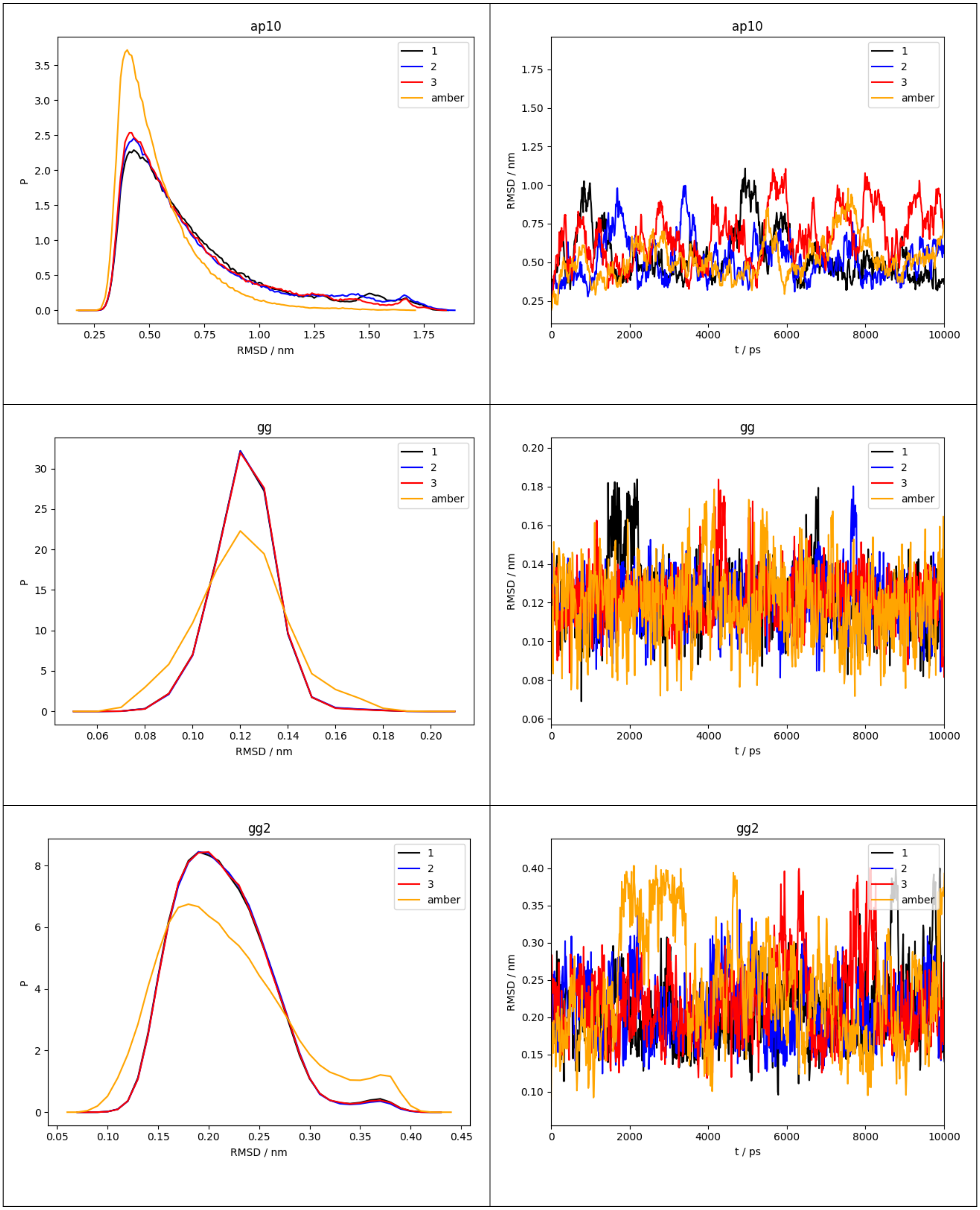
ap10
P
RMSD / nm
1
2
3
amber
ap10
RMSD / nm
t / ps
1
2
3
amber
gg
P
RMSD / nm
1
2
3
amber
gg
RMSD / nm
t / ps
1
2
3
amber
gg2
P
RMSD / nm
1
2
3
amber
gg2
RMSD / nm
t / ps
1
2
3
amber

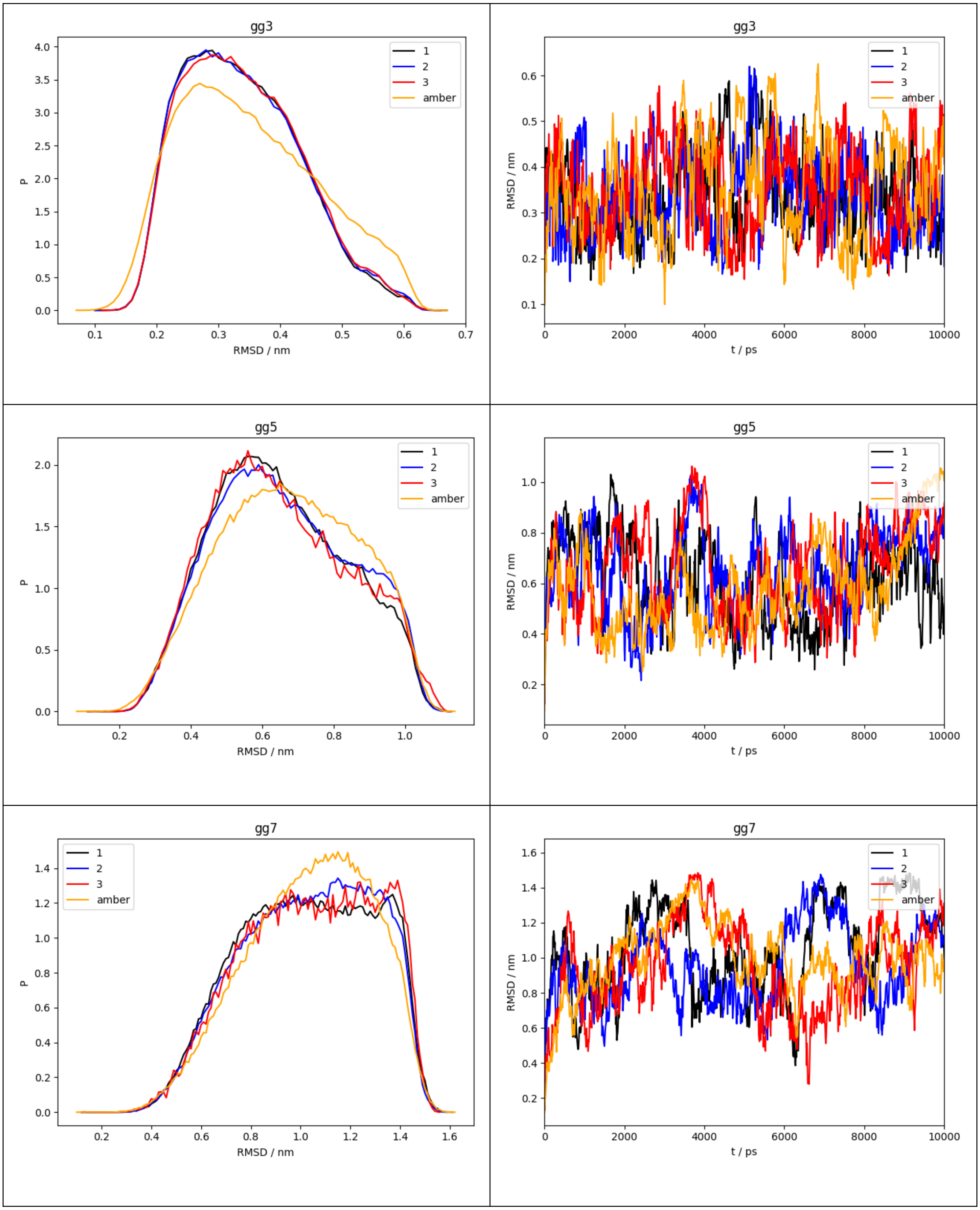
gg3
P
RMSD / nm
1
2
3
amber
gg3
RMSD / nm
t / ps
1
2
3
amber
gg5
P
RMSD / nm
1
2
3
amber
gg5
RMSD / nm
t / ps
1
2
3
amber
gg7
P
RMSD / nm
1
2
3
amber
gg7
RMSD / nm
t / ps
1
2
3
amber

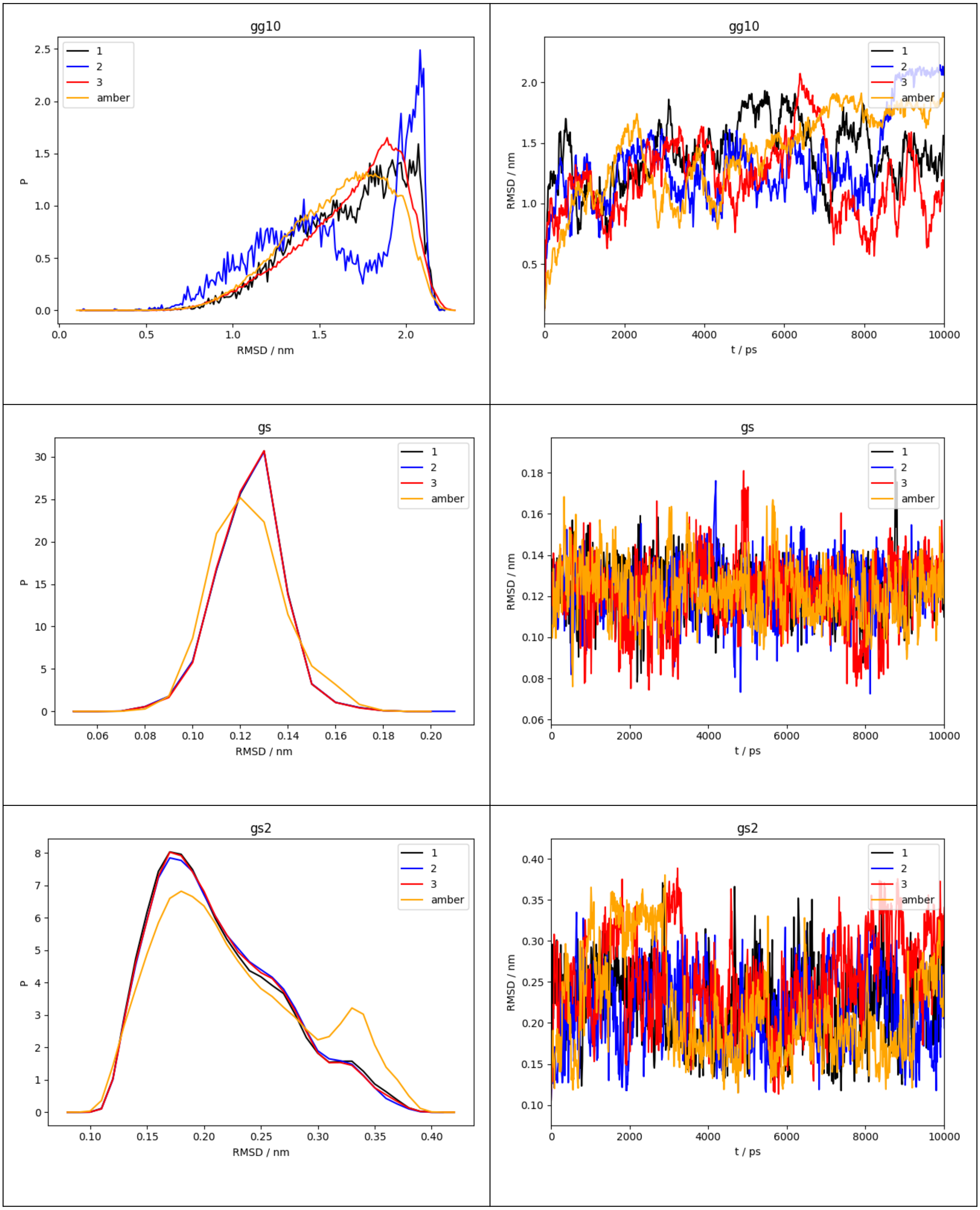
gg10
P
RMSD / nm
1
2
3
amber
gg10
RMSD / nm
t / ps
1
2
3
amber
gs
P
RMSD / nm
1
2
3
amber
gs
RMSD / nm
t / ps
1
2
3
amber
gs2
P
RMSD / nm
1
2
3
amber
gs2
RMSD / nm
t / ps
1
2
3
amber

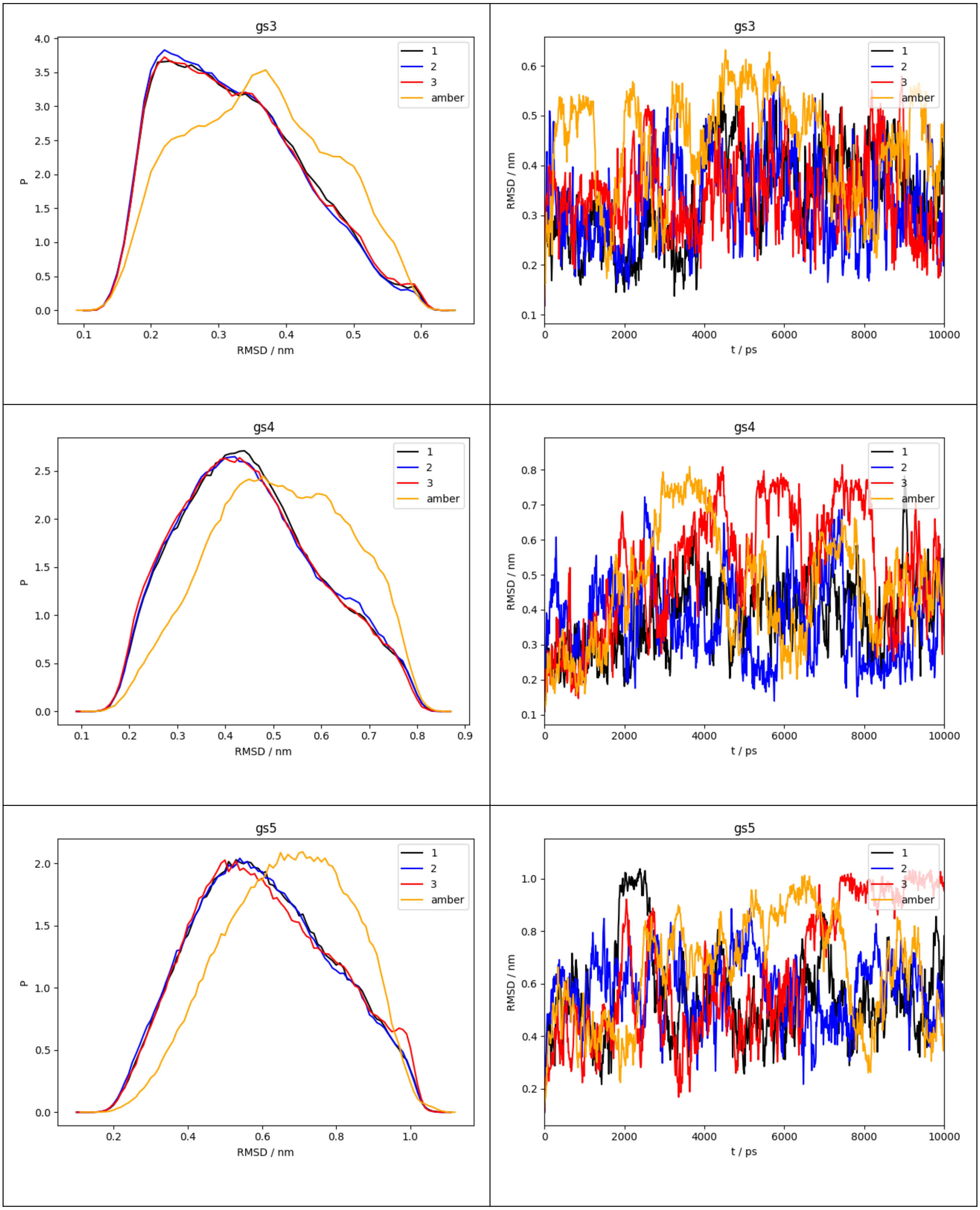
gs3
P
RMSD / nm
1
2
3
amber
gs3
RMSD / nm
t / ps
gs4
P
RMSD / nm
gs4
RMSD / nm
t / ps
gs5
P
RMSD / nm
gs5
RMSD / nm
t / ps

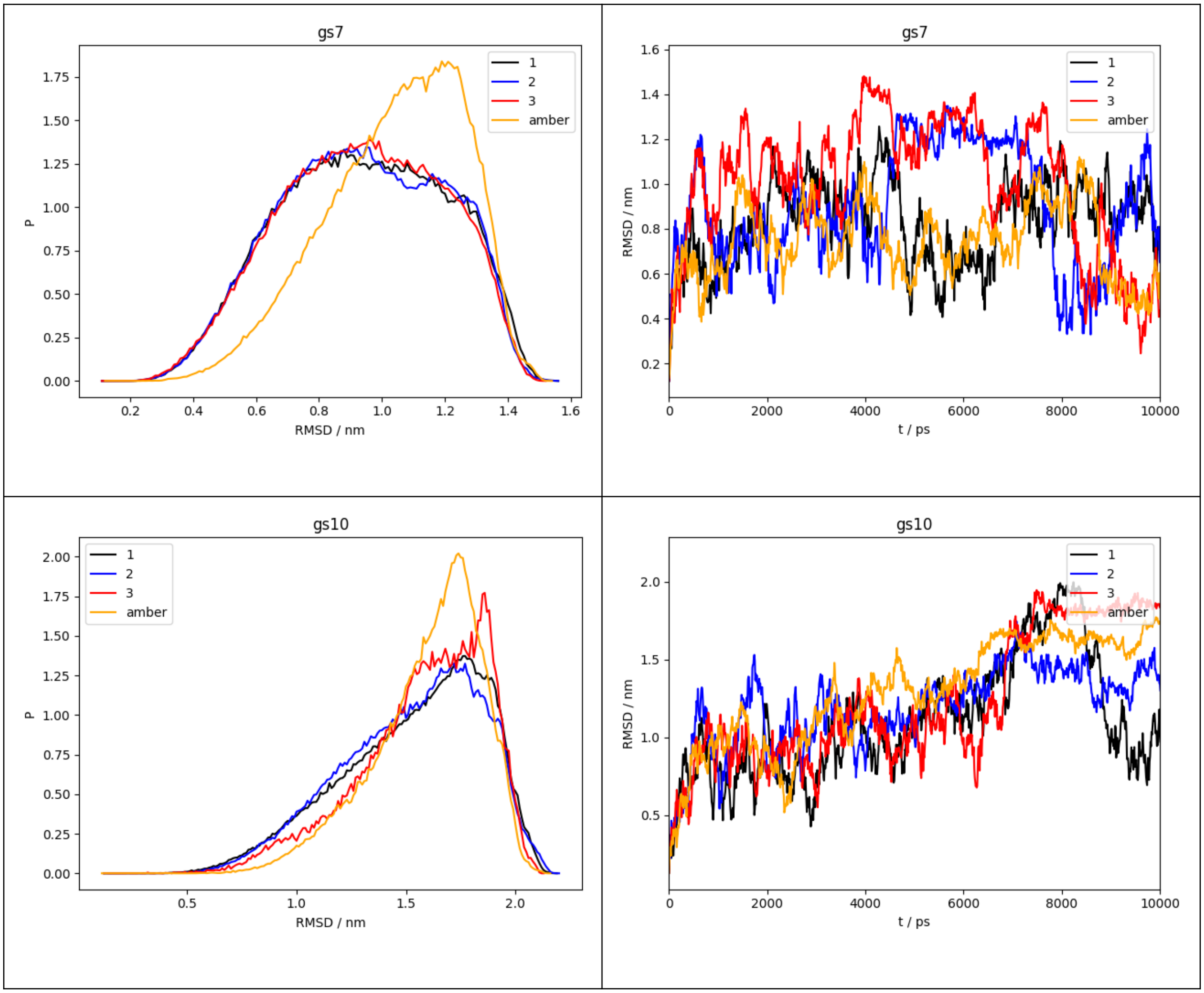
gs7
P
RMSD / nm
1
2
3
amber
gs7
RMSD / nm
t / ps
gs10
P
RMSD / nm
gs10
RMSD / nm
t / ps

Figure S1 - (left) Backbone RMSD distributions during the three replica trajectories (different color curves) of MD simulations using CHARMM36m forcefield and the single replica using ff14SB forcefield. (right) RMSD during the first 10 ns trajectory time in all the replicas

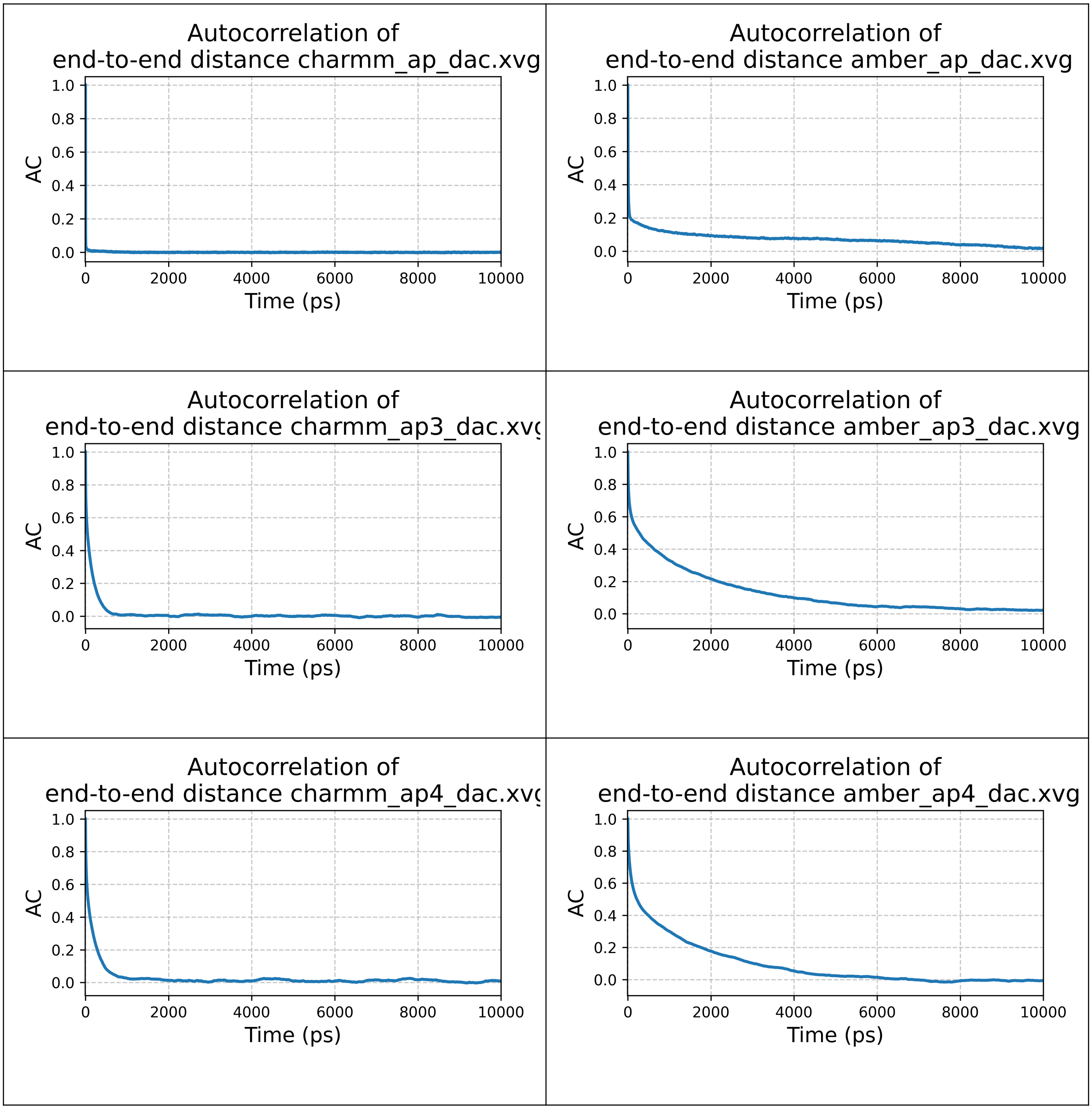
Autocorrelation of
end-to-end distance charmm_ap_dac.xvg
AC
Time (ps)
Autocorrelation of
end-to-end distance amber_ap_dac.xvg
AC
Time (ps)
Autocorrelation of
end-to-end distance charmm_ap3_dac.xv
AC
Time (ps)
Autocorrelation of
end-to-end distance amber_ap3_dac.xvg
AC
Time (ps)
Autocorrelation of
end-to-end distance charmm_ap4_dac.xv
AC
Time (ps)
Autocorrelation of
end-to-end distance amber_ap4_dac.xvg
AC
Time (ps)

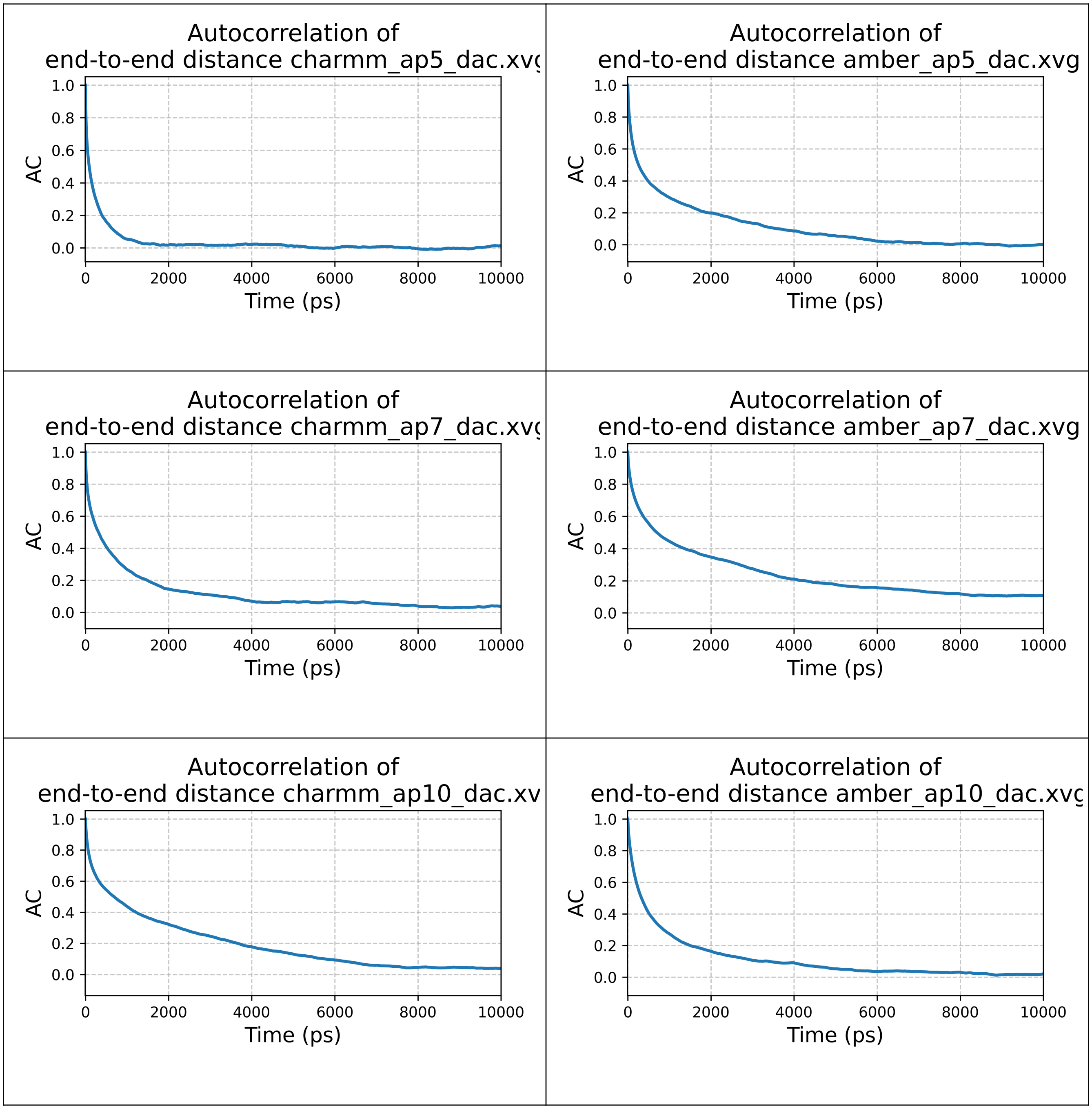

Autocorrelation of end-to-end distance charmm_ap5_dac.xvg
Autocorrelation of end-to-end distance amber_ap5_dac.xvg
Autocorrelation of end-to-end distance charmm_ap7_dac.xvg
Autocorrelation of end-to-end distance amber_ap7_dac.xvg
Autocorrelation of end-to-end distance charmm_ap10_dac.xv
Autocorrelation of end-to-end distance amber_ap10_dac.xvg
AC
Time (ps)
0 2000 4000 6000 8000 10000
0.0 0.2 0.4 0.6 0.8 1.0

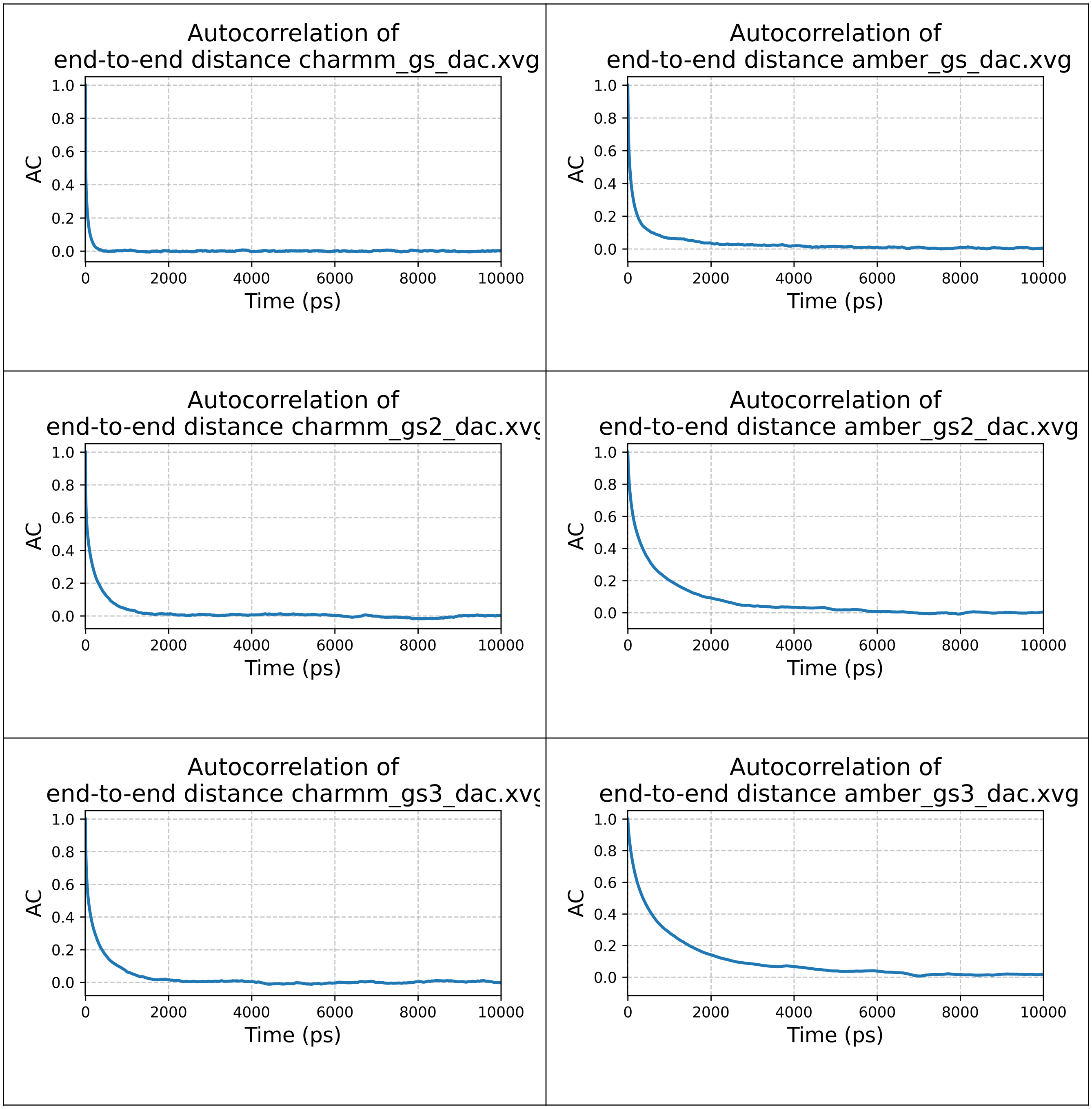
Autocorrelation of
end-to-end distance charmm_gs_dac.xvg
AC
Time (ps)
Autocorrelation of
end-to-end distance amber_gs_dac.xvg
AC
Time (ps)
Autocorrelation of
end-to-end distance charmm_gs2_dac.xv
AC
Time (ps)
Autocorrelation of
end-to-end distance amber_gs2_dac.xvg
AC
Time (ps)
Autocorrelation of
end-to-end distance charmm_gs3_dac.xv
AC
Time (ps)
Autocorrelation of
end-to-end distance amber_gs3_dac.xvg
AC
Time (ps)

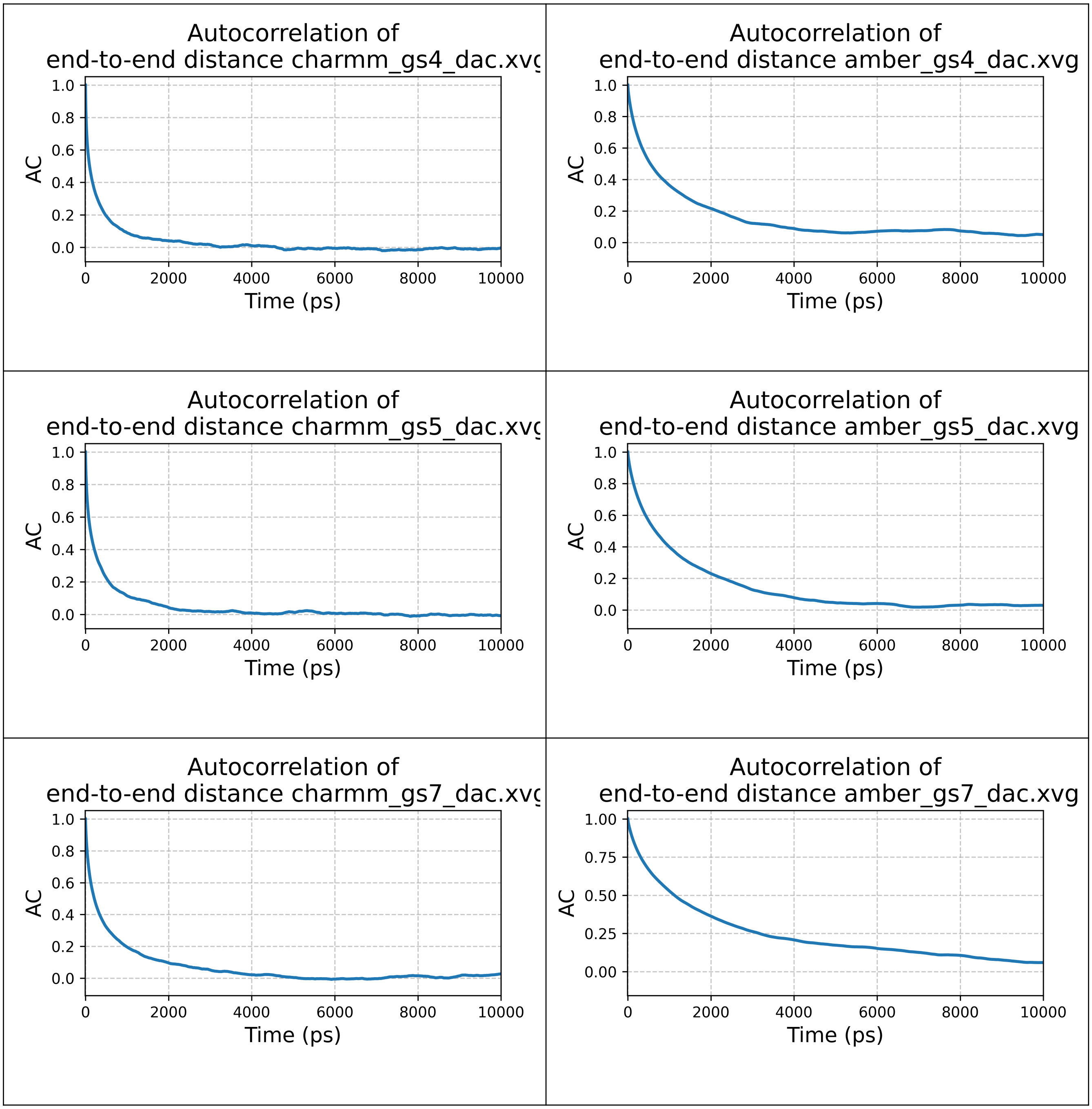
Autocorrelation of
end-to-end distance charmm_gs4_dac.xv
AC
Time (ps)
Autocorrelation of
end-to-end distance amber_gs4_dac.xvg
AC
Time (ps)
Autocorrelation of
end-to-end distance charmm_gs5_dac.xv
AC
Time (ps)
Autocorrelation of
end-to-end distance amber_gs5_dac.xvg
AC
Time (ps)
Autocorrelation of
end-to-end distance charmm_gs7_dac.xv
AC
Time (ps)
Autocorrelation of
end-to-end distance amber_gs7_dac.xvg
AC
Time (ps)

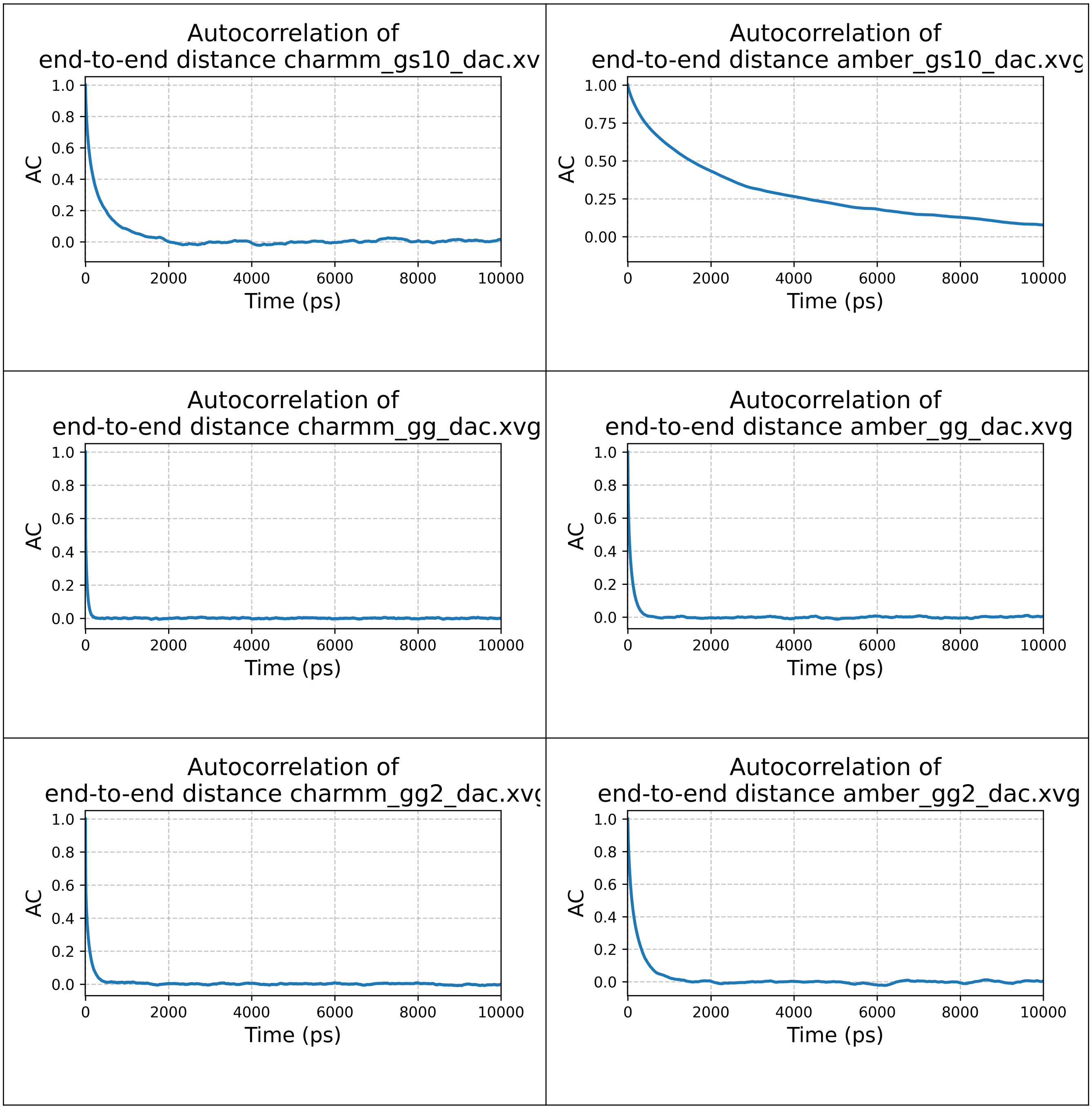
Autocorrelation of
end-to-end distance charmm_gs10_dac.xv
AC
Time (ps)
Autocorrelation of
end-to-end distance amber_gs10_dac.xvg
AC
Time (ps)
Autocorrelation of
end-to-end distance charmm_gg_dac.xvg
AC
Time (ps)
Autocorrelation of
end-to-end distance amber_gg_dac.xvg
AC
Time (ps)
Autocorrelation of
end-to-end distance charmm_gg2_dac.xvg
AC
Time (ps)
Autocorrelation of
end-to-end distance amber_gg2_dac.xvg
AC
Time (ps)

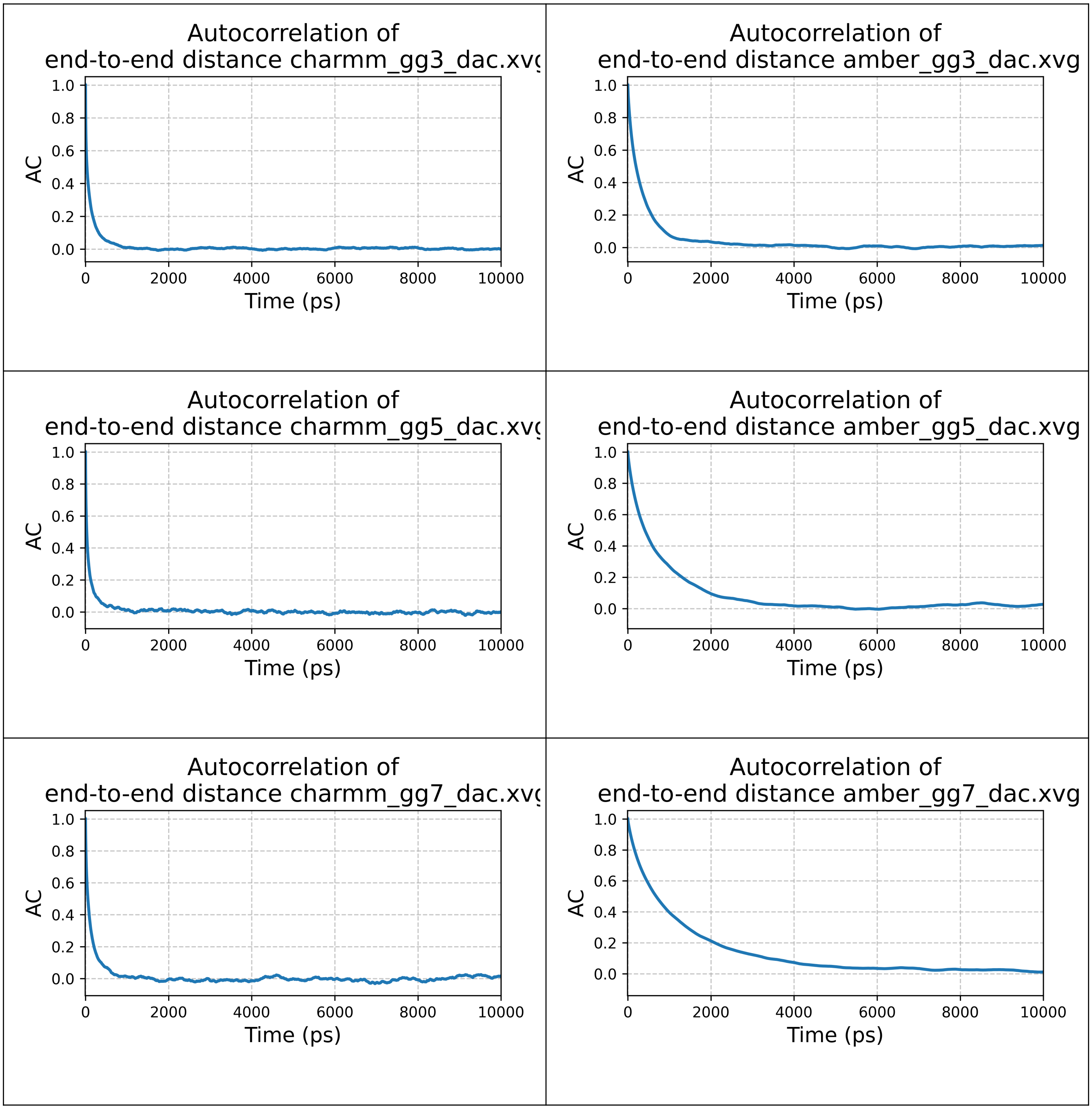
Autocorrelation of end-to-end distance charmm_gg3_dac.xv
Autocorrelation of end-to-end distance amber_gg3_dac.xvg
Autocorrelation of end-to-end distance charmm_gg5_dac.xv
Autocorrelation of end-to-end distance amber_gg5_dac.xvg
Autocorrelation of end-to-end distance charmm_gg7_dac.xv
Autocorrelation of end-to-end distance amber_gg7_dac.xvg
AC
Time (ps)
0.0 0.2 0.4 0.6 0.8 1.0
0 2000 4000 6000 8000 10000

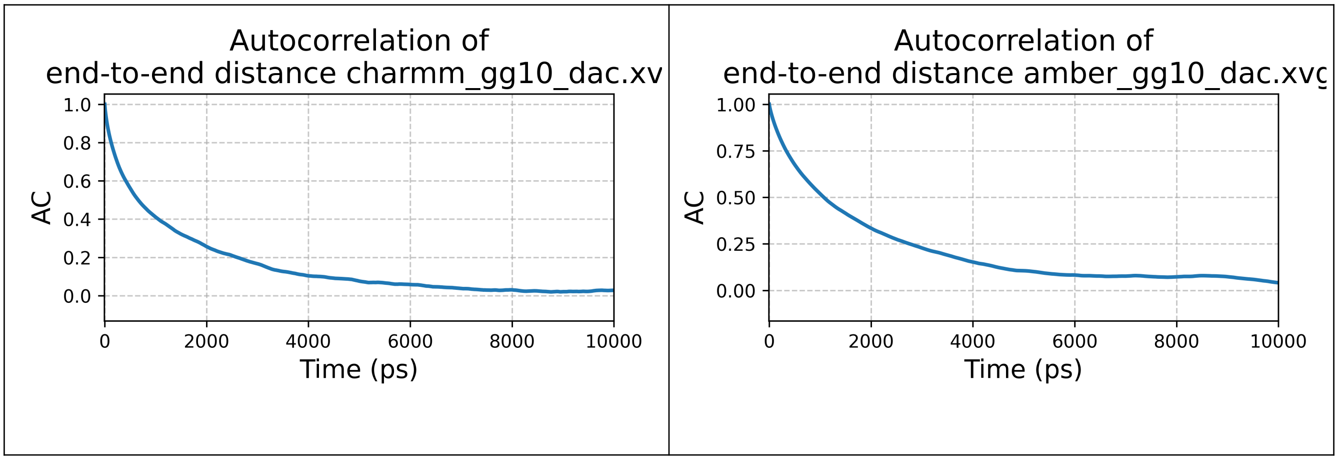

Figure S2 - Autocorrelation functions of End-to-End distances for linkers simulated in CHARMM36m (left column) and ff14SB + OPC (right column). Shown are times for the first 10000 ps of each of the trajectories.
